# A hundred and two just-so stories: exploring the lay evolutionary hypotheses of the manosphere

**DOI:** 10.1017/ehs.2025.10020

**Published:** 2025-10-09

**Authors:** Louis Bachaud, Macken Murphy, Sarah E. Johns

**Affiliations:** 1 Université de Lille, Lille, France; 2 University of Kent, Canterbury, UK; 3 University of Melbourne, Melbourne, Australia; 4 Kent and Medway Medical School, Canterbury, UK

**Keywords:** Evolutionary hypotheses, just-so stories, manosphere, evolved sex differences, misogyny

## Abstract

The manosphere is a collection of online antifeminist men’s groups whose ideologies often invoke Darwinian principles and evolutionary psychological research. In the present study, we reveal that the manosphere generates its own untested and speculative evolutionary hypotheses, or ‘just-so stories’, about men, women, and society. This is a unique phenomenon, where lay (i.e. non-expert) individuals creatively employ evolutionary reasoning to explain phenomena in accordance with their particular worldview. We thus assembled the first dataset of lay evolutionary just-so stories extracted from manosphere content (*n* = 102). Through qualitative analysis, we highlight the particularity of the manosphere’s lay evolutionism. It is a collective bottom-up endeavour, which often leads to practical advice and exhibits a male gender bias. We further show that 83.3% of manosphere just-so stories pertain to sex differences and that only 36.3% explicitly signal that they are speculative. Given this evidence that lay communities collectively engage in evolutionary hypothesizing, we reflect on implications for evolutionary scholars and for the field as a whole in terms of ethics and public image. Lastly, we issue a call for renewed discussion and reflection on evolutionary hypothesizing, a central yet somewhat neglected feature of evolutionary behavioural science.

## Graphical abstract

**Figure:**
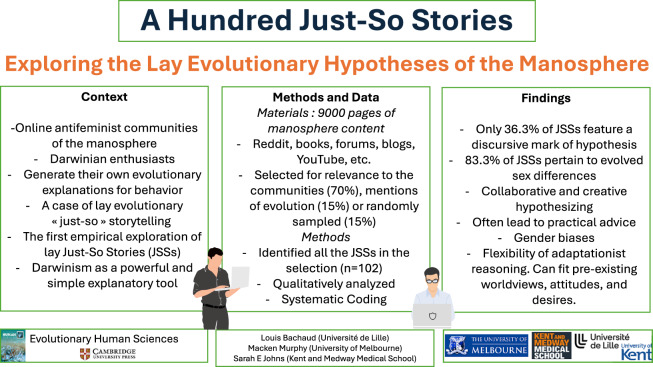

## Social media summary

This study examines a data set of manosphere-made evolutionary hypotheses and reflects on implications for scientists.

## 1. Introduction

Recently, evolutionary scholars have voiced concerns about the appropriation of their research by the online antifeminist communities of the manosphere (Conroy-Beam, 2024). Two online researchers have even recently included evolutionary psychology (EP) in their definition of the manosphere: ‘The manosphere is an online network of disparate and sometimes conflicting, sometimes overlapping assemblages. These formations are united in their antipathy toward feminism, their reliance on evolutionary psychology, and their belief that Western civilization is under threat’ (Ging & Murphy, 2021). In the present paper, we adopted a typology common in manosphere research, subdividing the manosphere into five communities: Men’s Rights’ Activists (MRAs), Pickup-Artists (PUAs), The Red Pill (TRP), Men Going Their Own Way (MGTOW), and incels (e.g. Krendel *et al*., 2022; Ribeiro *et al*., 2021). The defining features and ideology of each community are summarized in Supplementary Material S1.

Our study focuses on the second item of Ging and Murphy’s (2021) definition: the manosphere’s enthusiasm for Darwinian evolution, and in particular for EP. Because these communities have a focus on gender relations and sex, they routinely draw inspiration from EP research on human mating and evolved sex differences. According to recent research, this evolutionary psychological research is often enlisted to support misogynistic beliefs through the addition of value judgements, exaggerations, or selective biases (Bachaud, 2025; Bachaud & Johns, 2023; Baselice, 2023). However, the manosphere’s enthusiasm for evolution goes beyond appropriation and selective interpretation of existing research.

In this paper, we present evidence that the manosphere also generates its own attempts at evolutionary hypotheses. Indeed, the basics of evolutionary reasoning are not particularly difficult to grasp, and have been widely popularized by talented science writers (e.g. Dawkins, 1976; Pinker, 2002). Thus, it is not surprising that laypeople (i.e. non-experts) use this Darwinian lens to explain the world around them. We refer to these lay Darwinian-inspired explanations as ‘just-so stories’ to differentiate them from established hypotheses.

In evolutionary science, the ‘just-so story’ phrase was popularized by paleontologist Stephen Jay Gould. In a 1978 critique of sociobiology, he wrote: ‘When evolutionists study individual adaptations, when they try to explain form and behaviour by reconstructing history and assessing current utility, they also tell just-so stories – and the agent is natural selection’ (Gould, 1978). While paleontologists like Gould rely mostly on the fossil record, or nowadays on genetic evidence, which are material products of evolutionary history, behavioural scientists have to rely on hypotheses concerning the potential past adaptive functions of behaviour (Tooby & Cosmides, 1990).

Nowadays, this process of hypothesis generation has proved so fruitful that it has spawned many fields, such as evolutionary medicine (Nesse, 2019) or psychology (Buss, 2024), causing an ardent defender of the method to rejoice: ‘Evolutionary psychologists and other adaptationist researchers have rightly come to ignore the just-so story slur that Gould attempted to apply to entire fields of study’ (Alcock, 2018). Yet, the ‘just-so story’ phrase is a useful reminder that evolutionary explanations are easy to produce, but difficult to validate empirically.

Addressing that fact, evolutionary scholars have argued that the ‘difference between a just-so story and a well-accepted adaptationist explanation is the amount of direct evidence available’ (Olson & Arroyo-Santos, 2015). However, this criterion does not allow for precise categorization: for example, how much evidence is needed for a just-so story to be considered a legitimate hypothesis? How should different types of evidence be weighed? Moreover, in the evolutionary behavioural sciences, different hypotheses about the evolution of a trait compete, with some rising and falling out of favour (Lloyd, 2005).

Here, we argue that even a hypothesis that has been widely dismissed should not be considered as a just-so story. Indeed, as long as the researchers who proposed it at the time provided empirical evidence, which was accepted in a peer-reviewed publication, even subsequent disproval does not change the nature of the hypothesis. Otherwise, it would be fair to assume that the passing of time will prove most of the current content of evolutionary behavioural sciences to have been just-so stories, as the scientific enterprise of falsification continues (Popper, 1959). This would be quite an unjust reflection on the scientists who rigorously produce adaptationist hypotheses and empirical tests.

The operational definition of ‘just-so story’ adopted in this paper is therefore the following: a hypothetical evolutionary explanation for behaviour whose predictions have not been rigorously tested so as to warrant publication in a scientific outlet. It is henceforth abbreviated as ‘JSS’.

In the present study, we present and systematically review a dataset of 102 different JSSs produced by online lay manosphere communities. These communities are united by a focus on heterosexual relationships and evolved sex differences, two themes which are also typical of EP (e.g. Buss, 2023). In the manosphere, established hypotheses from EP literature circulate alongside untested lay speculations. Examining these is an excellent way to shed light on the form and content of the crude avatars of EP which circulate in popular culture and lay audiences (McCaughey, 2008).

One of the original concerns behind Gould’s coining of ‘just-so story’ was the possibility of devising a seductive (but unsubstantiated) evolutionary explanation for just about any trait or behaviour. In the manosphere, prevailing views of women vary, but are almost uniformly negative, and would fulfil any reasonable definition of sexism. Women are portrayed alternatively as ruthlessly manipulative or as emotionally immature, as supremely cunning or as incapable of logical thinking (for extensive analysis of manosphere ideologies, see Bachaud, 2025). The present review of manosphere JSSs shows how Darwinian reasoning can be enlisted to reinforce this gender ideology (for a similar appropriation by nineteenth-century US feminists, see Hamlin, 2014). The existence of this competing body of lay JSSs also raises questions for evolutionary scholars, which we address at the end of this paper.

## 2. Data and methods: qualitative analysis of manosphere discourse

The material for this paper comes from an extensive qualitative analysis of manosphere discourse (Bachaud, 2025; Bachaud & Johns, 2023). For this analysis, materials spanning 30 years of the manosphere’s mostly online history were selected (1993–2023), including various sources such as forum threads, Reddit posts, books, e-books, blog posts, web articles, online encyclopedia entries, tweets and YouTube videos. The corpus consisted of 70% content central to the studied communities, either identified as such by other scholars, or because of online popularity metrics such as citations, views, or upvotes (for the complete list of corpus materials and rationale for inclusion, see Supplementary Material S2). Another 15% of the corpus consisted of randomly sampled discussions on Reddit and forums, to include everyday discussions among manospherians (details of the sampling procedure can be found in Supplementary Material S3). The last remaining 15% was selected ad hoc over five years of browsing the manosphere in search of relevant content mentioning Darwinian evolution (corpus constitution and content selection are summarized in Supplementary Material S4). After transcription of audio and video content, the final corpus represented 9,000 pages of discourse (see Supplementary Material S2 for list of materials), evenly divided between the five manosphere branches.

To cite manosphere discourse, two different cases were established. For pseudonymous forum and Reddit users, anonymity and expectations of privacy are respected. Therefore, the citations do not provide hyperlinks towards the source material, and only the manosphere community, type of platform, and date will be provided (e.g. incel, Reddit, 2017). For public manosphere writers and content creators, the material is cited in the standard way. These measures were approved by the Universities of Kent and Lille’s review boards (approval numbers, respectively, 8-PGR-20/21 and QSMDC 2021-478-S91).

Analysis of the corpus was driven by a set of research questions on the role of evolutionary science within manosphere discourse and ideology. The questions pertained to the specific concepts and theories appropriated in manosphere ideology, the modalities, effects, and purposes of these appropriations, and the discrepancies between manosphere Darwinism and the scientific state of the art. To address these, relevant information from each document in the corpus was extracted and thematically categorized using a standardized template (for an example of completed template, see Supplementary Material S5).

### 2.1. Case studies on key manosphere just-so stories

As data analysis progressed, it became clear that the manosphere was producing its own evolutionary speculations. Some just-so stories in particular were foundational to their community’s ideology and constantly invoked, discussed, and refined. To illustrate this collective dimension of manosphere evolutionary storytelling, two foundational JSSs were selected as case studies, that is, those on the evolution of ‘approach anxiety’ and ‘shit tests’. They were thoroughly analysed and compared to the relevant academic literature.

### 2.2. Systematic review of manosphere just-so stories

To shed light on the unique phenomenon of manosphere evolutionary storytelling, we also effected a systematic review of all JSSs found in the corpus. However, based on our definition of JSS, identification was extremely difficult, as distinguishing existing hypotheses from lay-made JSSs requires exhaustive knowledge of evolutionary literature, which no individual presumably possesses. To address that issue, a three-step selection process was put in place.

First, the first author reviewed the entire discourse corpus, and extracted all the hypotheses that they did not recognize from the literature. Several criteria were adopted: the potential JSS should concern human evolution and behaviour, it should specifically include Darwinian reasoning (e.g. fitness, competition, selection, genes). Statements such as ‘Evolution made it that way’ or ‘that’s just evolution’ were therefore not included. When several occurrences of the same JSS were present in the corpus, it was counted as one, the goal being to review the contents of manosphere JSSs rather than their frequency or popularity. One hundred and twenty presumable JSSs were thus identified. For practicality and ease of comprehension (the manosphere has its own jargon, see Farrell *et al*., 2020), those were reformulated in more concise and scientific terms, recapitulating the core content of the JSS: which trait evolved and why (see recapitulated dataset in Supplementary Material S7).

Then, this preliminary dataset was reviewed by the other authors. Any hypothesis that they recognized from the literature was dismissed. When manosphere discourse resembled established hypotheses, it was determined on a case-by-case basis whether it differed from the literature, that is, whether the adaptation and/or evolutionary trajectory hypothesized were substantially different. As a result, 18 hypotheses were dismissed, leaving 102 presumable just-so stories.

Finally, analyses of the preliminary dataset were presented at the European Human Behaviour and Evolution Association (EHBEA) yearly conference in Montpellier, on 19 April 2024. At the end of the presentation, the evolutionary scholars in the audience were asked for help. They were given a QR code directing to the dataset and were asked to browse it and signal hypotheses they recognized from the literature. No additional hypotheses were dismissed as a result of this procedure (this three-step selection process is summarized in Supplementary Material S8). Thus, our dataset of 102 manosphere-made just-so stories was compiled. This is one of the major contributions of this study, as to our knowledge this is the first dataset of the kind. The original anonymized manosphere materials containing the JSSs are available in Supplementary Material S6, and the recapitulated spreadsheet dataset can be found in Supplementary Material S7.

Using both the original excerpts and the recapitulated dataset, the first and third authors then blindly coded each JSS for three binary criteria: evolved sex difference (1 = Yes, 0 = No); discursive marks of hypothesis (1 = Yes; 0 = No); and sexual selection (1 = Yes; 0 = No). To ensure that they were working with the same definitions, the first author provided a coding manual (reproduced in Supplementary Material S9). The second author then computed the Cohen’s *Kappa* interrater reliability statistic. Cases with coding disagreements were reconciled by the first and third authors on a case-by-case basis, until final percentages were reached (see Supplementary Material S10 for complete coding details). Finally, for comparison, a dataset of evolutionary hypotheses from the latest evolutionary psychology textbook was retrieved and coded by the first author only (Buss, 2024). No independent coding was deemed necessary because the hypotheses formulated in the textbook are unambiguously worded compared to lay just-so stories (see Supplementary Material S11 for procedure and dataset).

## 3. Qualitative case studies

### 3.1. Approach anxiety: a case of collaborative, bottom-up, lay hypothesizing

In his EP textbook, David Buss describes the ‘bottom-up’ (sometimes also called ‘observation-driven’) approach to evolutionary hypothesizing, which starts with everyday observations. ‘Once the observation is made about the existence of a phenomenon’, he explains, ‘we can then proceed in a bottom-up fashion and generate a hypothesis about its function’ (Buss, 2024). In bottom-up fashion, manosphere just-so stories often stem from everyday observations and experiences. This is manifest in PUAs’ fascination for what they call ‘approach anxiety’ and the way they speculate about it. Pickup-Artists or members of the ‘seduction community’ dedicate themselves to creating and applying conversational, behavioural, and lifestyle techniques to seduce heterosexual women. This community has a strong commercial nature, with the most influential PUAs offering paid tutoring sessions, group seminars, and selling dating guides (scholars have called it a ‘community-industry’: O’Neill, 2015).

In most PUA dating guidelines, the first step is usually called ‘approaching’, that is, going up to initiate conversation with a woman. However, most aspiring PUAs report extreme nervousness and anxiety during the approach. This ‘approach anxiety’ is one of the most discussed topics in PUA forums and guides. Year after year, newcomers to the community ask their more experienced peers about ways to circumvent this crushing nervosity. In one of the most influential dating guides of the community, the *Venusian Arts Handbook*, dating coach Erik Von Markovik, best known by the pseudonym ‘Mystery’, laid out his canonical JSS to explain the evolution of approach anxiety (JSS#29):

“Logically, rejection causes us no harm. But emotionally, rejection can be a punishing experience. To understand this, we must look at the ancient environment for which we were designed.

In a tribal group, there will be some small number of available women of breeding age. When a man approaches one, he risks rejection, and if that happens, all the other women will know, which will diminish his value in their eyes – maybe to the point where none of the women will mate with him. This is called preselection – women look for social validation of their choices. A suitor who is preselected will be more attractive, whereas a man who has been rejected will be less so.

Another factor regarding approach anxiety is the possibility that she may already be taken, in which case there is a component of real, physical danger to any male who approaches her.

For all these reasons and more, men are naturally selected to experience approach anxiety” (Mystery, 2007).

With no mark of hypothesis, nor any empirical test, Mystery thus confidently declares that approach anxiety is a male-specific adaptation that arose to answer two distinct selective pressures: loss of reputational status incurred by female rejection in small-scale mating environments and potential harm caused by another male’s mate guarding. Mystery believes this adaptation to be male-specific, arguing that ‘[m]en take a larger risk than women when first approaching. In ancient times, this posed a legitimate safety concern and thus men still experience *approach anxiety*’ (Mystery, 2007). Even if he does not use the term, he argues that this adaptation is an evolutionary mismatch in today’s Western dating world: ‘Logically, of course, modern society fixes these problems. If I am rejected, I can simply go to another part of the bar, or leave the bar entirely. I will probably never see any of those people again. But my emotions don’t know that. My emotions are trying to do what’s best for me’ (Mystery, 2007). This has great motivational value for readers of the *Venusian Arts Handbook*: their anxiety is depicted as the dysfunctional emotional legacy of a distant past that should be disregarded and overridden.

For years, this JSS remained a canonical feature of PUA guides and theories, and approach anxiety became a ubiquitous phrase in their discussions. Over time, it was collaboratively extended, refined, or challenged by the community. In fact, almost all its features have been criticized by other PUAs. For example, one argues that women experience even more approach anxiety than men (JSS#32): ‘I think that women have much stronger rejection anxiety than men do, which is why society has evolved such that men always have to approach’ (PUA, Reddit, 2016).

Others argue that approach anxiety is not a specific mating-related adaptation, but just caused by a generic fear of rejection (JSS#31):

“Back in prehistoric times a person being socially rejected and cast out on their own by their clan meant that you’d probably die alone in the wild. Those of us alive today are all descended from the people who managed not to be outcasts, or the outcasts who were able to find a clan that liked them enough to take them in. Those with a stronger fear of rejection were less likely to behave in such a way that would cause their clan to cast them out, and if that’s true then that explains why the fear of rejection is so prevalent” (PUA, Reddit, 2015).

Another PUA Redditor concurs and says that ‘Mystery’s theory is way to specific and complicated and acts like AA [approach anxiety] is some kind of unique mechanism’, while to him, ‘it seems far more likely […] that trying hitting on a girl is a situation that tends to bring out common anxieties like a fear of (any kind of) rejection’ (PUA, Reddit, 2015).

A member of the Red Pill community (a more politicized offshoot of the seduction community; see definition in Supplementary Material S1) argues that approach anxiety is actually a by-product of another psychological adaptation, coming from infants’ fear of separation from their mothers (JSS#55):

“My theory that I’d love to make a post on at some point is that a child’s greatest fear is abandonment/rejection from the parents since death is essentially guaranteed from this in the jungle. When a random woman rejects a male to the point where he becomes depressed, anxious, etc it’s because that male is because he subconsciously views that woman as his mother in a sense. That fear is a child survival mechanism projecting onto said woman” (TRP, Reddit, 2018).

Lastly, some PUAs question the adaptive approach altogether. One argues that Mystery misrepresents the selective pressures at work in ancestral environments:

“I don’t think people cold approached back then. In tribe settings of maybe 50-150 people, everyone most likely knows each other either through family ties or family history (your great-grandparents knew theirs). So this brings up two points:

1. You won’t get kicked out of the tribe that easily. Think of the times you upset your parents/family. Most likely you weren’t banned from seeing them.
2. Relationships/marriage were most likely arranged by the family or forced onto the woman. There was no need to cold approach and build attraction” (PUA, Reddit, 2017).

He therefore concludes that ‘AA comes from modern social conditioning. It’s not a natural thing’ (PUA, Reddit, 2017). Another Redditor also ridicules Mystery’s ‘baseless theory’: ‘It’s kind of like if he said: the reason job interviews make people nervous is that in caveman times if you tried to take someone else’s role/job in the tribe and did a worse job than them, you’d be killed by your community’ (PUA, Forum, 2015).

Mostly unbeknownst to the EP academic community, online groups of laypeople have for almost two decades been debating adaptive accounts for the evolution of approach anxiety. Fundamentally, their discussions have much in common with everyday debates among evolutionary scholars: is the mechanism domain-specific or domain-generic? Is it sex differentiated? What were the ancestral selective pressures? Could it not be a by-product? Is it an adaptation at all? The main difference, which is of course epistemologically crucial, is the absence of empirical tests, and of non-anecdotal evidence. Although the bottom-up approach to evolutionary hypothesizing is recognized as a perfectly legitimate scientific method (Buss, 2024), its proponents acknowledge that it should not stop at speculating about an observed behaviour’s function: ‘The bottom-up approach is completed when – and only when – the researcher then “turns around” and generates novel testable predictions based on this hypothesized psychological mechanism’ (Lewis *et al*., 2017). Thus, manosphere JSSs fall short of the standards of adaptationist bottom-up hypothesizing. However, the example of approach anxiety shows that the manosphere does not solely interpret and distort existing scholarship but also produces and maintains its own autonomous pseudo-Darwinian body of knowledge on specific issues, as further shown by the ‘shit test’ just-so story.

### 3.2. Shit tests: male-biased evolutionary just-so storytelling

The concept of ‘shit test’ is a cornerstone of the Red Pill view of female behaviour, underpinned by a speculative evolutionary narrative (JSS#45). In the context of heterosexual mating, shit tests are thought to be a woman’s way of evaluating a man’s mate value, as explained in this Reddit guide:

“What is a shit test?

Female attractiveness is clearly obvious to even the most casual of observers. Beauty, femininity, and approachability, the three pillars of female SMV [sexual market value], are all on display in any girl you can see, hear, and speak with.

Male attractiveness, being basically a rubric for ‘how useful would this guy be during a riot or zombie apocalypse?’, is less evident from casual interaction. While muscularity can be seen, wits, nerve, resourcefulness, persistence, and other behavioural qualities cannot.

Men can passively observe attractiveness, but girls must actively probe for it.

Enter the **shit test**, wherein a girl gives a man a hard time (‘some shit’) to see how well he copes with it.

This takes a number of different forms, and can be at pretty much any level of intensity, but if a girl suddenly does something that **seems intended to bother you, and is totally unprovoked**, you can be pretty sure you’re being shit-tested” (TRP, Reddit, 2018).

There is a remarkable consensus among Red Pillers about the adaptive function of these tests, which are sometimes called ‘fitness tests’, making their Darwinian-inspired nature even clearer. These ‘tests’ are supposed to gauge a man’s confidence, dominance, and resourcefulness, and thus, his suitability as a mate. This being the ultimate function of the behaviour, it does not have to be conscious, Red Pill ideologue Rollo Tomassi argues:

Women’s shit testing is a psychologically evolved, hard-wired survival mechanism. Women will shit test men as autonomously and subconsciously as men will stare at a woman’s big boobs. They cannot help it, and often enough, just like men staring at a nice rack or a great ass, even when they’re aware of doing it they’ll still do it. (Tomassi, 2013)

Surveying Red Pill texts, it seems that just about any statement or action directed by a woman towards a man in a context of heterosexual mating can be construed as a shit test. Very conveniently, Red Pillers use the supposed ultimate function of these tests to advise men to disregard anything women might say. Indeed, because shit tests are thought to be unconscious testing devices, their discursive content does not matter, only men’s reactions to them do. Thus, Red Pillers invariably recommend not to take shit tests seriously.

In fact, the best way to ‘pass’ them is usually to ignore them: *‘never forget that* **a shit test can be passed by literally any response which shows that you are not rattled**’ (TRP, Reddit, 2018). As with the approach anxiety JSSs review above, practical advice usually follows the formulation of the evolutionary story. Let us consider such advice from a Red Pill Reddit guide entitled ‘Everything you need to know about shit tests’:

“– The woman asks: ‘Do you have a girlfriend?’:

Translation: Are you a beta? (Can you get laid?) – The correct answer is always yes (it increases your preselection.) Women love poaching men from other women, they essentially find whatever is ‘in demand’ to be attractive, that’s what we refer to as ‘preselection’.

Ways to pass this test: ‘she told me not to tell anyone’ – ‘We’re not Facebook official’ – ‘I don’t cuddle her after sex, so no?’ (TRP, Reddit, 2014).

– The woman says: ‘I have a boyfriend!’:

Translation: I have Schrödinger’s boyfriend, demonstrate to me you’re high value and I’ll fuck you regardless. It is hilarious when they say this.

Ways to pass this test: ‘What boyfriend, your imaginary one?’ – Then laugh in her face. – ‘Sounds like you’re shit out of luck, I’m going to have to fuck your friend instead, feel free to watch’” (TRP, Reddit, 2014).

Evidently, Red Pillers tell men not to take what women say seriously. Even factual statements such as ‘I have a boyfriend’, which could be assumed to show a lack of sexual interest, are assumed to be false, and interpreted as invitations to ‘try harder’. Thanks to the evolutionary rationale behind the shit test concept, Red Pillers pose as authorities on female behaviour and tell their thousands of male readers to constantly disregard women’s words and concerns. In fact, according to them, this is exactly what women ultimately desire, as argued by Rollo Tomassi: ‘Women want to be told “No”, and constantly test a man’s resolve to say this to her (a.k.a. shit testing) in order to affirm that she’s made the right choice’ (Tomassi, 2013). This is a typical manosphere JSS: there are no marks of hypothesis, nor any non-anecdotal empirical evidence.

It is quite obvious that this JSS was devised by men. Indeed, nothing in sexual selection theory dictates that only women would be interested in evaluating a mate’s suitability (for a sex-neutral explanation of similar phenomena, see Zahavi, 1977). In fact, selecting a mate with a suitable attributes should have massive fitness benefits for both sexes (Buss, 2024; Stewart-Williams & Thomas, 2013). Following the Red Pill’s evolutionary reasoning, it seems quite unlikely that only women would have evolved psychological mechanisms to address this selective pressure. A second issue is that of demarcation: what exactly is a ‘shit test’? In Red Pill writings, the definition seems infinitely flexible, as any complaint or challenge can be framed as a shit test:

“[G]oing out on a date with a woman is a collection of shit tests ‘to see if you’re worth having sex with’. Being in a police interrogation room is a collection of shit tests. Being heckled by members of the audience as a comedian is a collection of shit tests. And it goes on and on and on. Shit tests are an inescapable and recurring element of life, so you better get good at handling them” (TRP, Reddit, 2014).

With so wide an application, the very concept of a simple, punctual ‘test’ loses most of its meaning. It seems more plausible that individuals (not just women) constantly assess others’ suitability as mates by considering the totality of their words and actions. If one were to imagine a serious (i.e. non-gender-biased and empirical) adaptationist research programme on ‘shit tests’, it would thus bear little resemblance to the Red Pill JSS. Such a programme would explore the conscious and unconscious cues people process to evaluate a mate’s character and suitability for a relationship. It might also investigate when and why individuals engage in behaviours designed to elicit specific responses from a potential partner, such as seeking reassurance about their interest or commitment. Many of the ‘shit tests’ described by Red Pill adherents appear to arise from genuine feelings of anxiety or concern. However, the Red Pill’s default response is to suggest that a woman’s anxiety can only be alleviated through dishonesty, aloofness, and dominance, as a way to unconsciously ‘reassure’ her of one’s fitness.

Although this approach may be effective in some contexts, this should be empirically demonstrated, and is likely influenced by factors such as personality, context, and relationship dynamics. Setting ethical considerations aside, significant cross-cultural evidence indicates that honesty and understanding are highly valued traits in a partner (Eastwick *et al*., 2025). This suggests that, more often than not, the most effective way to reassure a current or prospective partner may simply be to sincerely address their concerns, rather than dismissing them in the playful manner promoted by Red Pill advocates.

### 3.3. Discussion

In bottom-up fashion, manospherians use evolutionary reasoning and concepts from evolutionary psychology to explain their own emotions about women and their interactions with them. The first major difference with the relevant academic literature is the assertive confidence with which these explanations are voiced, despite their lack of empirical testing. We can compare manosphere JSSs on this issue with a PhD dissertation published in 2002 on the same phenomenon (Kugeares, 2002). Like Mystery, Kugeares argues that there might indeed be such a context-specific evolved anxiety mechanism, which she calls ‘dating anxiety’. Like him, she points out reputational costs incurred by rejection, as well as potential violence from other males as selective pressures. Unlike Mystery, she adds mating opportunity costs as another cost of romantic rejection. More importantly, she does not believe this to be a male-specific adaptation. In contrast with the manosphere, her hypothesis is clearly signalled as one (the ‘Mate Value Hypothesis’). Lastly, in rigorous adaptationist fashion, she carefully lays out the function of the mechanism and derives predictions which are then subjected to empirical testing.

Pickup-Artist just-so storytelling differs from the EP literature in another fundamental way. It is meant to be applied, as practical advice is invariably derived from those competing JSSs. For approach anxiety, because the trait is thought to be an evolutionary mismatch, this gives PUAs a potent rationale for ignoring the emotion they deem maladaptive. It must be noted, however, that is entirely possible to devise practical applications from evolutionary psychological research. It would require that the adaptationist hypotheses lead to robust (i.e. replicated and cross-cultural) empirical findings, which would then be used to guide clinical/public policy interventions or individual behaviour (see advocacy for positive evolutionary psychology in Geher & Wedberg, 2019). In that case, however, these interventions would not be guided by a pseudo-Darwinian fitness-maximizing agenda, as in the Red Pill, but simply by the empirical findings derived from adaptationist hypothesizing. In fact, some evolutionary psychologists have advocated for confidence training for men who are anxious in dating contexts (Li *et al*., 2020).

Through the ‘shit test’ JSS, Red Pillers advise thousands of readers to repeatedly disregard the content of women’s words, and advocate for evasiveness or plain dishonesty. This sort of pseudo-Darwinian gender paternalism was also present in the early PUA seduction guides of the 2000s, such as Mystery’s, where readers were given biology-based reasons to circumvent sexual consent and ignore women’s reluctance to engage in sex (see Denes, 2011). JSS#57 of the manosphere dataset illustrates that aspect of manosphere writings. The author warns his readers against ‘asking a girl to kiss, or asking if you can touch her boobs in bed’ to signal respect of ‘feminism and consent’. Indeed, he argues that this would signal a ‘lack of social proof and dominance’, leading women to infer that ‘you have bad genes’, thus prompting rejection (TRP, Reddit, 2020). Here, manosphere Darwinism implies amorality, as (speculative) fitness becomes the only relevant criterion to guide action.

These case studies showcase ubiquitous features of manosphere JSSs. First, manospherians collectively devise bottom-up evolutionary explanations for gender relations and female behaviour. Second, manospherians confidently assert such explanations for behaviour, without acknowledging their speculative nature. Third, these JSSs concern evolved sex differences, one of the core aspects of manosphere ideology. Lastly, these laypeople often seem to gravitate towards evolutionary explanations featuring sexual selection. Those last three features were quantified through independent coding of the JSS dataset and results are presented below (see all coding details in Supplementary Material S10).

## 4. Systematic review

### 4.1. Results

Each JSS was coded, as shown by the example of JSS#9 in Table 1.

**Table 1.** Example of a reformulated and coded manosphere just-so story

JSS#	Behaviour/Trait	Fitness Benefits/Evolutionary History	Sexual Selection (1 = Yes; 0 = No)	Evolved Sex Difference (1 = Yes; 0 = No)	Marks of Hypotheses (1 = Yes; 0 = No)
9	Women have more pleasure from sex than men do, even though they often pretend the opposite	If this greater propension for pleasure had not evolved, it is hard to fathom why women would engage in sex, and endure the risks and pains of pregnancy, childbirth, and child-rearing	0	1	0

Raters had good agreement on whether a given JSS contained the discursive marks of a hypothesis (Cohen’s *Kappa* = 0.73, *p* < 0.001) and whether it focused on evolved sex differences (Cohen’s *Kappa* = 0.60, *p* < 0.001). Raters discussed to reach final agreement, ultimately finding that 36.3% of manosphere JSSs (37/102) contained marks of hypothesizing, and 83.3% of JSSs (85/102) pertained to evolved sex differences. The JSS dataset did not significantly differ from the textbook dataset on that variable (2 × 2 nonparametric chi-square test, χ^2^ = 3.1036, *p* = 0.08), although hypotheses on sex differences seemed marginally more common in the JSS dataset. Despite trying to unambiguously define sexual selection in our coding manual (see Supplementary Material S9), the agreement between raters on which JSSs pertained to sexual selection or not was fair (Cohen’s *kappa* = 0.32, *p* < 0.001) but not substantial, so we removed this from further analysis.

### 4.2. Discussion

To assess how many manosphere JSSs could be clearly identified by lay readers as speculative, we coded for the presence or absence of discursive marks of hypotheses (e.g. ‘maybe’ or ‘I guess’; see Supplementary Material S9 for coding manual). With only 36.3% of manosphere JSSs featuring some mark of hypothesizing, the most common scenario for laypeople browsing the manosphere is therefore to encounter confident and assertive evolutionary narratives – one of the major differences between lay JSSs and the evolutionary behavioural sciences.

At first glance, the manosphere’s lay evolutionism seemed extremely focused on the evolution of sex differences. Yet, this is one of the hallmarks of academic evolutionary psychology, too. We thus compared and found no significant difference between the share of hypotheses on sex difference in the JSS dataset and in the latest EP textbook, whose author is a leading proponent for the study of evolved sex differences (Buss, 2024). This focus on evolved sex differences should therefore be regarded as a signature of both academic EP and of its manosphere rendition – and as a potential explanation for the popularity of EP in the manosphere, as manosphere ideologies tend to insist on a starkly differentiated view of the sexes (Bachaud, 2025).

Lastly, it proved difficult to find agreement between raters on which JSSs pertained to sexual selection or not. Indeed, sexual selection is a special case of natural selection, and the boundary between the two can be unclear (Mayr, 1972). For example, accruing resources can both directly contribute to natural selection of an organism’s phenotype, as well as make the organism outcompete intrasexual reproductive rivals, thus favouring its sexual selection. This blurriness was compounded by the fact that the data consisted in lay JSSs, whose wording was often casual or imprecise (see original excerpts in Supplementary Material S6).

## 5. General discussion

Sociologist of science Ullica Segerstråle argues that ‘evolutionary biology is a surprisingly flexible field’, which ‘may be employed to prove almost any point one wishes’ (Segerstråle, 2014). Manospherians themselves seem to be aware of this, as shown by JSS#90, which appears to be a satirical example of an absurd just-so story about evolved female preference for odorous male faeces.

The unique manosphere JSSs dataset presented here reveals the ease with which evolutionary reasoning can be used to justify one’s desires, actions, and ideas, especially in the absence of empirical tests. In the examples reviewed above, the JSSs conveniently espouse manospherians’ intentions. Pickup-Artists want to ‘approach’ all women they find desirable in public spaces: a JSS tells them to ignore their anxiety, which is thought to just be a legacy from ancestral times, mismatched to the contemporary environment (JSS#29). Red Pillers would like to evade or ignore women’s challenging or embarrassing questions: a JSS tells them that this is exactly what women unconsciously want (JSS#45). As for explicitly seeking out sexual consent, a JSS makes sure that this is rendered unnecessary, and even undesirable (JSS#57).

As argued by Barrett (2023), evolutionary explanations have a particularly ‘seductive allure’, insofar as they provide complete explanations for given observations, from the ultimate to the proximate levels. Yet, as he also points out, these explanations are more likely to be false, as they hinge on being correct both about proximate phenomena and their evolutionary origins. In the case of the manosphere JSSs, there is little doubt that most of them are probably false. First, they are clearly made by men for men, and thus feature some unmistakable male biases: why wouldn’t women feel anxious at approaching attractive strangers, given the potential risks? And why would only women be testing for a partner’s worth? Couldn’t men be hypothesized to conduct ‘tests’ to evaluate women’s suitability as mothers, for example?

Second, these JSSs hinge on an extremely demeaning view of female nature with little resemblance to known reality. This can be easily confirmed by browsing some of the alleged sex differences in the dataset. For instance, it contains three different separate JSSs explaining women’s supposed intellectual inferiority (JSS#7, JSS#16, and JSS#41). More generally, it seems than evolutionary hypothesizing is made to support any negative belief or stereotype about women: ‘Women are incapable of sexual fidelity’ (JSS#10); ‘Men are less likely than women to abuse positions of power’ (JSS#12); ‘Women have a less developed sense of personal responsibility’ (JSS#48); ‘Women are scatterbrained, and have lower capacity for focused attention’ (JSS#59); ‘Men are better than women at most tasks’ (JSS#69; JSS#72). While there can be little doubt about the gender-biased, and speculative nature of these JSSs, their very existence raises a complex question: should evolutionary scientists care about this alternative set of evolutionary explanations for behaviour and society? Examining the context where these JSSs were collected suggests so.

### 5.1. Implications for the evolutionary science community

In manosphere discourse, JSSs seamlessly mingle with legitimate hypotheses from the literature. Considering how challenging it was, even for three evolutionary scholars, to identify and separate manosphere-made JSSs from published hypotheses (see Methods section), this task cannot be expected of any layperson browsing the manosphere. As a consequence, people encountering manosphere discourse might equate it with evolutionary behavioural sciences in general.

This risk is accentuated by the propensity of manospherians to cite peer-reviewed articles, even though those do not test for the specific JSSs they forward. See for example JSS#77, found on the incel’s own wiki platform (for analysis of incels.wiki, see Roser *et al*., 2023). Summarizing a study that found that vegetarian men are perceived by women as less attractive (Timeo & Suitner, 2018), curators of the incel wiki explain that this female preference might have been selected because of the nutritiousness of meat brought by male hunters over evolutionary history (‘Scientific Blackpill’, n.d.). Yet, they do not signal that this evolutionary hypothesis does not feature in the study they cite (which focuses on the masculine gender role): it is a just-so story.

This use of scientific citations undoubtedly grants credibility to manosphere ideas, as explained by a former Red Piller to a British newspaper: ‘The movement’s use of evolutionary psychology convinced my rational mind that everything I read was a scientific fact suppressed by feminists’ (Tait, 2017). Whether one is an enthusiast or a critic of the manosphere, a partial glance at manosphere content suffices to equate manosphere ideology with evolutionary psychology, a confusion encouraged by manospherians themselves. For example, incels.wiki features a page called ‘The Scientific Blackpill’, which makes heavy use of academic citations, and is framed as a literature review of EP: ‘The Scientific Blackpill is about understanding the nature of human social and sexual behaviour with a particular focus on evolutionary psychological perspectives’ (‘Scientific Blackpill’, n.d.).

More worrisome for the field’s reputation is the fact that other scholars studying the manosphere also make that equation between academic EP and manosphere ideology. For example, in a 2018 critique of the Red Pill’s main’s subreddit (r/TheRedPill), sociologist Shawn van Valkenburg argues that Red Pill ideology ‘faithfully incorporates contemporary EP literature into its philosophy’, and calls for critical investigation of ‘the extent to which a branch of academia is implicated in r/TRP’s construction’ (Van Valkenburgh, 2018; for a critical investigation of Van Valkenburgh’s claims, see Bachaud, 2025, p. 386).

Although recent analyses from within have concluded that evolutionary psychology has entered a mature phase of Kuhnian ‘normal science’ (Barrett, 2020), the field remains contested and controversial (see O’Neill, 2016). The existence of an alternative and unsubstantiated body of EP-like analyses is bound to stoke these controversies, especially considering the wide circulation of manosphere discourse. For example, Mystery and his JSSs are given pride of place in the bestselling memoir *The Game* (Strauss, 2005), while Rollo Tomassi has been popularizing the notion of shit tests and other JSSs through his successful *Rational Male* book series (Tomassi, 2013, 2015, 2017).

Beyond those reputational stakes for EP as an academic discipline, there is evidence that these JSSs have a concrete impact on the behaviour of their adherents. This is revealed by Neil Strauss’s book *The Game*, where he recounts his life with dating coach Mystery, arguably the most influential popularizer of Darwinism in the manosphere. Strauss recalls that evolutionary psychology stoked Mystery’s jealousy towards his ex-partner’s new boyfriend, giving ‘an intellectual justification for his antisocial emotions and his desire to harm the organism that had mated with his woman’ (Strauss, 2005). When his friends attempted to get a depressed Mystery into therapy, Strauss recalls, they were ‘dismissed with a long-winded explanation of how his [Mystery’s] emotions and actions were evolutionarily justified’. In her ethnographic immersion among the London PUA community, sociologist Rachel O’Neill was in fact struck by the ‘*frequency* and *ease*’ with which evolutionary ‘imperatives’ were invoked by her interviewees: ‘Those who employed this repertoire were utterly convinced of their own evolutionarily ordained need for sex and felt compelled to explain why they and other men must conduct their intimate lives in accordance with such biological dictates’, she wrote (O’Neill, 2018).

These examples reflect the naturalistic fallacy, the moral justification of an action because it is seen as ‘natural’ and biologically determined. Evolutionary psychologists have repeatedly criticized this fallacy, arguing that no moral judgement should be derived from the adaptive nature of a given trait or behaviour (Pinker, 1997, 2002). Anyone familiar with EP literature is bound to have encountered warnings about this fallacy. However, justifications for behaviour in the manosphere seem to be opportunistic and motivated rather than simply guided by the sole belief that what is ‘natural’ is intrinsically desirable. In fact, when alleged evolved psychological mechanisms are seen as undesirable, as in the case of approach anxiety, then manosphere writers tell their readers to go ‘against their nature’ (e.g. Mystery, 2007). What seems to matter most here is whether the action is seen as desirable or expedient in the first place, which then receives post hoc justifications that either commit the naturalistic fallacy (e.g. in the case of Mystery’s personal conduct) or not (e.g. in the case of approach anxiety).

Our examination of the approach anxiety and shit test JSSs reveals that evolutionary storytelling in the manosphere is often a prelude to prescriptive behavioural and lifestyle advice. Given the content of those JSSs, one might reasonably expect this advice to have harmful consequences for women’s autonomy and sexual consent. While evolutionary scholars cannot be held responsible for these behaviours, they are uniquely poised to address this conundrum. First, as experts on academic literature, they are the most qualified to help separate legitimate empirical research from unsubstantiated storytelling, which is one of the main achievements of the present study. Second, because they know how serious adaptationist thinking and research should be conducted, they are capable of identifying gender-biased pseudoscience, as shown by our critique of the ‘shit test’ JSS. Lastly, evolutionary scholars working on human behaviour are also used to the statistical, ethical, and epistemological implications of evolutionary behavioural research, such as the reminders that mean tendencies do not necessarily allow for inference on individual cases, that explaining is not justifying, and that normative judgements should not be derived from the fact that something is an adaptation – reminders that are often absent from the manosphere avatar of EP.

There has recently been a nascent trend in science popularizers and professionals critically comparing manosphere beliefs to academic literature (e.g. Alexander, 2022, 2023; Bachaud, 2025; Bachaud & Johns, 2023; Baselice, 2023; Conroy-Beam, 2024). In the book *Hack Your Mating: An Evolutionary Psychologist’s Guide to a Life of Sexual Abundance*, evolutionary scholar Antonios Vakirtzis provides his own prescriptive dating advice based on EP (2018). Interestingly, he addresses ‘approach anxiety’, a concept coming from Pickup-Artist literature, and expresses surprise ‘that behavioral scientists, and particularly evolutionary psychologists, have not bothered with the topic’ (Vakirtzis, 2018). Then, he goes on to provide his own hypothesis about approach anxiety. To him, ‘approaching’ an unknown woman triggers mechanisms that evolved to prevent ancestral men from courting outgroup women, which he argues was more costly and dangerous than courting ingroup women. However, without empirical tests nor peer review for this hypothesis this is yet another just-so story. This example shows that manosphere JSSs can provide a source of inspiration for evolutionary scholars, something we discuss next.

Our manosphere JSS dataset contains 102 evolutionary hypotheses. Note that we did not attempt to evaluate the likelihood that these were in any way valid, which would require designing and conducting hundreds of specific empirical studies. This raises the question: should evolutionary psychologists in fact rigorously test the hypotheses forwarded by manospherians in an attempt to falsify them? On an epistemological level, the value of a hypothesis should not be judged by its origin, but by its resistance to falsification attempts (Popper, 1959). Therefore, there is no fundamental issue with having some of these JSSs being empirically tested by professionals. However, there are two potential risks in pursuing this avenue further. First, scientists’ time and resources are limited. We can doubt whether the best use of that time is to investigate hypotheses whose proponents exhibit such blatant gender biases and motivated thinking. Lastly, given the reputational risks discussed above, evolutionary scholars might want to avoid giving the impression that the manosphere is setting their research agenda.

Paradoxically, hypothesis generation is rarely discussed among evolutionary scholars, even though it is one of the most distinctive features of the field (guidelines are available, e.g. Lewis *et al*., 2017; Hunt & Jaeggi, 2025, but it remains to be seen how often these are followed). There are countless seminars on research methods or ethics, but relatively little attention is paid to training new scholars for rigorous adaptationist hypothesis generation. Maybe because this is a creative activity, which is implicitly assumed to be less prone to standardization and should be left to individuals. Anthropologist Martha McCaughey also argues that evolutionary reasoning is simply ‘fun’ and ‘can be a satisfying intellectual exercise’ (McCaughey, 2008).

The creativity and enthusiasm of manospherians for evolutionary explanations reveal that this satisfaction is not limited to professional scientists. Given this evidence that anyone can formulate an evolutionary story about anything, scientific adaptationism needs to clearly demarcate itself from lay evolutionism. By being honest about the research process and always carefully signalling hypotheses as such, by avoiding cavalier and overconfident statements about evolution of human behaviour, and more importantly by engaging in collective reflection, exchange, and training on hypothesis generation, evolutionary scholars would both distance themselves from lay pseudo-Darwinian speculations and make their science better.

### 5.2. Limitations and future research

As reflected in this paper’s title, this research should be considered exploratory, as there was no prior existing scholarship on this specific type of object: lay evolutionary hypotheses. Moreover, the manosphere data for this study were extremely noisy and heterogeneous, ranging from casual Reddit conversations to book-length exposés. In the process of reformulating these into a more manageable form, myriads of somewhat arbitrary linguistic decisions had to be made. Likewise, in the selection process, some judgement calls had to be made whereby genuine JSSs might have been discarded because they closely resembled peer-reviewed and published hypotheses (i.e. false negatives).

Although we strove to be thorough, it is also possible that at least a few of the JSSs in our dataset were mislabelled as just-so stories, and that they are in fact peer-reviewed and published hypotheses (i.e. false positives). Theoretically speaking, it is simply impossible to prove that something has never been published (absence of evidence is not evidence of absence). We hope that the publication of this dataset will pique the curiosity of other evolutionary scholars, who might in turn recognize some hypotheses therein (Supplementary Material S7). If so, we kindly encourage them to signal this to the corresponding author. Ultimately, our definition of JSS was meant as an operational definition for the needs of the empirical study, but the issue of delineating scientific from non-scientific content is an enduring conundrum in philosophy of science, which cannot be expected to be ‘solved’ anytime soon, if ever.

The present study revealed the propensity of manospherians to engage in evolutionary hypothesizing. Yet, three important dimensions of the phenomenon cannot be evaluated through discourse analysis alone. The first one concerns the process of hypothesis generation and could be investigated by interviewing manosphere content creators (such as Mystery, whom we failed to reach for an interview). Are those JSSs even consciously devised as such, or are they unconscious deformations of academic hypotheses? What evidence do manospherians browse before forwarding them? How does scientific knowledge circulate in these communities? And so on.

A second key dimension is the extent to which these JSSs are taken seriously. For all we know, given the common lack of marks of hypothesis, some of these JSSs might not be considered hypothetical at all. It does seem that their most adamant popularizers, such as Mystery, really believe in the evolutionary stories they tell. On the other hand, some manospherians are more critical, such as the Redditor who reminded others that approach anxiety was just ‘a theory pick up artists invented based on zero evidence’ (PUA, Reddit, 2017). It would therefore be necessary to evaluate manospherians’ intuitive feelings of certainty (FOC) about their just-so stories, and whether this certainty could be affected by debunking attempts from evolutionary psychologists. As shown by research in science education, FOC can indeed have an influence on the acceptance of given scientific beliefs, potentially explaining why some people with relatively high science literacy refuse to accept evolutionary theory (Ha *et al*., 2012).

Lastly is the most crucial element to investigate. Does adherence to these stories about the evolution of sex differences really impact these men’s actions, in particular towards women? Qualitative ethnographic evidence seems to suggest so, but experimental designs are necessary to cement this claim. Unfortunately, manospherians tend to be extremely hostile towards academia (Sugiura, 2021), and no experiments have ever been conducted on this population, to the best of our knowledge. Through priming and vignettes, studies have investigated potential inferences and normative judgments triggered by evolutionary explanations for behaviour. Dar‐Nimrod *et al*. (2011) found that reading evolutionary explanations for sex crimes did not make respondents more acceptant of these crimes, whereas sociocultural explanations made participants more condemnatory and punitive. Nettle *et al*. (2023) found that non-experts tend to make an incorrect inference: when they are presented with biological explanation for behaviour, they intuitively assume it is less malleable than behaviour explained through psychological or sociocultural factors. Such designs could be adapted to identify links between beliefs and behaviour among manospherians, for example by assessing whether exposure to gender-biased JSSs triggers changes in attitude towards women.

## 6. Conclusion

The basic principles of evolutionary reasoning are easy to grasp and have been widely popularized by gifted science communicators. In the manosphere, online antifeminist men’s communities employ this reasoning to make sense of the world around them, in particular concerning gender and sex differences. Exploring a dataset of manosphere-made just-so stories revealed unwarranted discursive confidence about speculative assertions, and a suite of male biases. These Darwinian-inspired speculations, which seem to be regarded by some of their adherents as absolutely true, do not obey the rules of scientific adaptationism. Given their disciplinary expertise, we believe that evolutionary scholars are uniquely equipped to critique these speculations, which borrow from EP’s credibility to advance a depreciatory view of women, while harming the public reputation of the field at large and in academia. Lastly, we see this as a call for renewed discussion and reflexivity on the hypothesis-generating process in the evolutionary behavioural sciences, which should not be seen as a ‘black box’ or purely creative endeavour, but be based on transparent and replicable principles for clear demarcation with lay pseudoscience and for better research.

## Supporting information

Bachaud et al. supplementary material Bachaud et al. supplementary material

## Supplementary material

The supplementary material for this article can be found at https://doi.org/10.1017/ehs.2025.10020.

## Research transparency and reproducibility

The entire corpus material is considered to be ‘personal data’ under GDPR regulations. In order to retrieve, store, and cite it, a GDPR derogation had to be filed on our behalf by the University of Lille’s Data Protection Officer under the provisions of GDPR’s article (submission no. DPO 2021-34). According to the law’s principle, the material is only to be stored for the duration of the study and cannot be shared with third parties. For the systematic review of manosphere just-so stories, the original JSSs are provided in Supplementary Material S6, the recapitulated version of the dataset in Supplementary Material S7, the coding manual in Supplementary Material S9, and all qualitative codes in Supplementary Material S10. The EP textbook dataset is provided in Supplementary Material S11. Lastly, the script used for data analysis is available on Supplementary Material S12.
