# Supplementary material for: A hundred and two just-so stories: exploring the lay evolutionary hypotheses of the manosphere
Source: Evol Hum Sci. 2025 Oct 9;7:e41. doi: 10.1017/ehs.2025.10020 (PMC12645320; doi:10.1017/ehs.2025.10020)
Supplement: Bachaud et al. supplementary material [file S2513843X25100200sup001.zip › S2513843X25100200sup001/Supplementary Material S1.pdf]

Supplementary Material S1: Description of Manosphere Groups

Group	What is the social reality for men in modern society?	What maintains or explains this social reality?	What would be a better social reality?	How can we achieve this better reality?
**Men's Rights Activists (MRAs)**	Men face gender-specific issues which are constantly ignored or downplayed in society. For example: the falling educational achievement of boys, or the higher rates of suicide and addiction among men.	A combination of (1) traditional cultural values which sees men as disposable; and (2) modern feminism, which systematically puts blame on men, and denies the existence of men's issues.	An egalitarian society, where every structural issue would be given its due attention. One where men would feel free to express their feelings and perspectives without fear of dismissal.	Recruitment; indignation; mobilization; activism; legal support and advice for men; creating counter-narratives; advocating for policy change.
**Pickup-Artists (PUAs)**	Most heterosexual men have a very hard time when it comes to dating, sex, and relationships. They live in a state of sexual and romantic inhibition and frustration.	Confidence issues and lack of experience, which make men anxious and self-conscious when interacting with women. This is compounded by their idealization of women.	Pickup-Artistry (also called "game") is a self-improvement method meant to allow men to satisfy their sexual and romantic expectations. It does not have a structural social agenda *per se.*	Fashion and grooming; studying social and sexual dynamics; developing an interesting personality; gaining experience through trial-and-error; concrete seduction tips and techniques.
**The Red Pill (TRP)**	Most men are "blue pilled," i.e., they put women on pedestals, accept feminism, and are unable to satisfy their own desires. They are ideologically conditioned to be "beta males," providing resources for women and society.	(1) Female nature is hypergamous, and women always strategize to extract benefits from men (money, support, validation).<br><br>(2) Feminism is a vast brainwashing enterprise that demonizes men and legitimizes all female behavior.	The Red Pill teaches men how to become "alpha": self-sufficient, masculine men, who pursue their own desires and interests, and reject socially imposed expectations. It helps men navigate current society, with no blueprint for an alternate society.	(1) "Taking the Red Pill": i.e., rejecting feminism; studying social and sexual dynamics; reading.<br><br>(2) becoming "alpha": bodybuilding; "game"; developing an independent and self-sufficient lifestyle.

**Men Going Their Own Way (MGTOW)**	MGTOW agree with the diagnoses of both MRAs and TRP. Western society is irremediably stacked against men. In particular, they have no legal protection against false rape allegations, no reproductive rights, and no say in matters of child support and custody.	(1) Feminism is the main culprit. It has seeped into education, governments, and become culturally hegemonic.<br>(2) Women's hypergamous nature, shallow and exploitative of men.<br>(3) Traditional expectations for men in careers, marriage, courtship, etc.	MGTOW are very pessimistic regarding Western society. They do not think it possible for men to challenge feminist hegemony, nor to defend their interest collectively. They thus advocate for men to individually check-out from social expectations, and romantic relationships.	Going one's own way: i.e., avoiding sexual and romantic interactions with women; developing a self-sufficient lifestyle; personal growth, satisfying one's own desires; investing in traveling, career or hobbies rather than in dating and relationships. Taking the red pill.
**Incels**	Society is stratified according to looks. Good-looking men (Chads) get the social and sexual validation. Regular looking men (normies), spend their lives working in the hope of attracting and providing for one woman (betabuxxing). Ugly men are at the bottom of society, they receive no attention nor validation.	(1) Lookism is ubiquitous. Social media and dating apps are shallow and reinforce it.<br>(2) Women ignore and behave cruelly towards ugly and awkward men.<br>(3) Feminists and other Social Justice Warrior (SJWs) ignore or ridicule incels' plight by spreading the male oppression myth.	There is no common social agenda. Some wish for a traditional society, with enforced monogamy and arranged marriages. Some call for sexual subjection of women. However, as a rule, incels tend to be hopeless and nihilistic, and rarely discuss concrete measures or a better society.	(1) Some incels try to "ascend" and escape inceldom through plastic surgery, bodybuilding, sex tourism, or sex workers.<br>(2) Some accept their situation, and cope with it through video games, drugs, pornography, etc.<br>(3) A minority turns towards suicide or mass violence.

The table above is reproduced from Bachaud, 2025. It describes the five manosphere branches through philosopher Kenneth Clatterbaugh's framework. In *Contemporary Perspectives on Masculinity: Men, Women, and Politics in Modern Society* (1990), Clatterbaugh introduces four questions that provide a "logical structure" to describe and critique men's movements:

“- 1. What is the social reality for men in modern society?
- 2. What maintains or explains this social reality?
- 3. What would be a better social reality?
- 4. How can we achieve this better reality?”[1]

[1] Clatterbaugh, Kenneth. (1990). *Contemporary Perspectives on Masculinity: Men, Women, and Politics in Modern Society*. Westview Press.
