# Supplementary material for: A hundred and two just-so stories: exploring the lay evolutionary hypotheses of the manosphere
Source: Evol Hum Sci. 2025 Oct 9;7:e41. doi: 10.1017/ehs.2025.10020 (PMC12645320; doi:10.1017/ehs.2025.10020)
Supplement: Bachaud et al. supplementary material [file S2513843X25100200sup001.zip › S2513843X25100200sup001/Supplementary Material S11.pdf]

Evolutionary Hypotheses in Buss, 2024

Hypothesis	Sex Difference	Developed on page
Sperm competition hypothesis (for sperm volume/testes size)	Yes	p.49
Disease avoidance hypothesis	Yes	p.66-67
Embryo protection hypothesis	Yes	p.68
Cooking hypothesis	No	p.69
Antimicrobial hypothesis	No	p.70
Frugivory byproduct hypothesis	No	p.71
Hunting hypothesis	No	p.71-72
Provisioning hypothesis	Yes	p.72
Show-off hypothesis	Yes	p.73
Gathering hypothesis	Yes	p.74
Savanna hypothesis	No	p.78
Evolved suicide adaptation hypothesis/Burdensomeness-to-kin hypothesis	Yes	p.88
Maladaptive by-product hypothesis (for suicide)	No	p.88
Male choice hypothesis	Yes	p.119
Female fertility hypothesis	Yes	p.136
Mating effort hypothesis	Yes	p.140
Sexual competition hypothesis (for eating disorders)	Yes	p.145
Kin altruism hypothesis (for male homosexuality)	Yes	p.146
Paternity confusion hypothesis	Yes	p.162
Status-enhancement hypothesis	Yes	p.163
Sexy son hypothesis	Yes	p.163
Mate expulsion hypothesis	Yes	p.163
Short-term for long-term goals hypothesis	Yes	p.163
Resource accrual hypothesis	Yes	p.164
Mate switching hypothesis	Yes	p.164
Good genes hypothesis	Yes	p.166-167
Mate skill acquisition hypothesis	Yes	p.168
Paternity uncertainty hypothesis	Yes	p.179
Mating opportunity cost hypothesis	Yes	p.179
Primary caretaker hypothesis	Yes	p.188
Attachment promotion hypothesis	Yes	p.188
Fitness threat hypothesis	Yes	p.188
Healthy baby hypothesis	Yes	p.191
Trivers-Willard hypothesis	No	p.193
Grandmother hypothesis	Yes	p.208
(Differential) grandparental investment hypothesis	Yes	p.218-220
Absent father hypothesis	Yes	p.220
Costly signaling hypothesis (for altruistic acts)	No	p.244
Alliance hypothesis	No	p.249
Status elevation hypothesis	Yes	p.261
Crazy bastard hypothesis	Yes	p.268
“Slip-up” hypothesis	Yes	p.281
Mate deprivation hypothesis	Yes	p.295
Sperm competition hypothesis of partner rape	Yes	p.296
Bodyguard hypothesis	Yes	p.297

Frequentist hypothesis	No	p.345
Social gossip hypothesis	No	p.348
Social contract hypothesis	No	p.348
Scheherazade hypothesis	No	p.348
Ecological Dominance/Social Competition hypothesis	No	p.349
Deadly innovations hypothesis	No	p.350
Display hypothesis	Yes	p.372

Methods: virtually every study in the evolutionary behavioral sciences tests one or several competing hypotheses, each pertaining to the evolutionary history and potential fitness benefits of a given trait or behavior. Recording all the hypotheses in an evolutionary psychology textbook would therefore be a Sisyphean task. A proxy for this is to record all the hypotheses which are explicitly named, such as the "grandmother hypothesis" which posits that menopause could have evolved in women if the fitness benefits of grandmaternal care to grandchildren outweighed those of continued fertility in old age. All the unique nominal compounds in the text (i.e., excluding references) ending in "hypothesis" were retrieved (n=57). For each one, it was determined whether the hypothesis implied a sex difference using the coding manual (Supplementary Material S8).

Data Cleaning: Five hypotheses were excluded. One is the "structural powerlessness hypothesis/sex role socialization/gender economic inequality" hypothesis (p.112) because it comes from the social sciences and is not based in evolutionary theory. Another is the "belief hypothesis" or "double-shot hypothesis" because it is a non-evolutionary hypothesis from social psychology (p.300-301). And the other three—the "predator confusion" and "inclusive fitness" and "parental investment" hypotheses—because they exclusively concern non-human animals (squirrels on p.210).

Aggregated features of these hypotheses are summarized in Table S1 below.

Table S2: Prevalence of Hypotheses in (Buss, 2024) relative to Sex Differences

**Characteristics of Evolutionary Hypotheses**		**Cases (%)**
Sex Difference	Yes	37 (74%)
	No	15 (26%)
