# Supplementary material for: A hundred and two just-so stories: exploring the lay evolutionary hypotheses of the manosphere
Source: Evol Hum Sci. 2025 Oct 9;7:e41. doi: 10.1017/ehs.2025.10020 (PMC12645320; doi:10.1017/ehs.2025.10020)
Supplement: Bachaud et al. supplementary material [file S2513843X25100200sup001.zip › S2513843X25100200sup001/Supplementary Material S2.pdf]

Supplementary Table S2: Manosphere Corpus Material

**Section**	**Group**	**Source/Author**	**Document**	**Nature**	**Grounds for Inclusion**[1]	**Pages**[2]
Central Content (CC)	The Red Pill (TRP)	r/TheRedPill	The Red Pill Sidebar	Web Article	Community/Academic	248
CC	TRP	Rollo Tomassi	*The Rational Male*	Book/E-Book	Community	286
CC	TRP	Illimitable Men	"The Red Pill Constitution".	Web Article	Community	13
CC	TRP	Ian Ironwood	*The Manosphere: A New Hope for Masculinity*	Book/E-Book	Community	282
CC	TRP	Puerarchy	All articles from the website's history	Web Article	Community	53
CC	TRP	Dalrock	"Top Posts" section	Blog Post	Community/Popularity	22
CC	TRP	The Private Man	Top 10 most popular posts of all time	Blog Post	Popularity – by comments	15
CC	TRP	Return of Kings	"Top 35 Most Important Articles on RoK"	Web Article	Community/Academic /Popularity	142
CC	TRP	r/TheRedPill	Most popular post of the subreddit, with comments	Reddit Post	Popularity – by upvotes Academic/Community	143
Random Sample (RS)	TRP	r/TheRedPill	Randomly sampled posts, with comments	Reddit Post	Random Draw	279
Other Related Material (ORM)	TRP	r/TheRedPill	Relevant Selection	Reddit Post	Relevant	151
ORM	TRP	TRP.RED	Relevant Selection	Web Article	Relevant	38
ORM	TRP	The Rational Male	Relevant Selection	Blog Post	Relevant	6
ORM	TRP	The Private Man	Relevant Selection	Blog Post	Relevant	13
ORM	TRP	Gynocentrism.com	Relevant Selection	Web Article	Relevant	6
ORM	TRP	Return of Kings	Relevant Selection	Web Article	Relevant	59

[1] The different labels in this columns are explained below this table.
[2] Number of pages after the original material was retrieved, archived, and video content transcribed, into PDF/Word form.

CC	MGTOW	r/MGTOW	All the MGTOW content from the subreddit's sidebar	Mixed[3]	Community	253
CC	MGTOW	r/MGTOW	Top 15 popular posts of all time, with comments	Reddit Post	Popularity – by upvotes	415
CC	MGTOW	MGTOW.com	All articles from the articles section	Web Article	Academic/Community	25
CC	MGTOW	MGTOW.com	Pinned pages	Mixed[4]	Academic/Community	72
CC	MGTOW	MGTOW.com	Two threads from the "Most Popular" section	Forum Thread	Academic/Community /Popularity	132
CC	MGTOW	SandMan	Five most popular videos	YouTube Video	Popularity – by views	110
CC	MGTOW	Anonymous	"The MGTOW manifesto"	Web Article	Community/Academic	3
CC	MGTOW	r/MGTOW.2.0	Top 5 popular posts of all time, with comments	Reddit Post	Popularity – by upvotes	82
CC	MGTOW	Anonymous	*The Masculine Principle*	Book/E-Book	Random Draw	198
RS	MGTOW	MGTOW.com	Randomly sampled threads	Forum Thread	Random Draw	78
RS	MGTOW	r/MGTOW	Randomly sampled posts, with comments	Reddit Post	Random Draw	92
RS	MGTOW	r/MGTOW2.0	Randomly sampled posts, with comments	Reddit Post	Random Draw	91
RS	MGTOW	Goingyourownway	Randomly sampled threads	Forum Thread	Random Draw	6
RS	MGTOW	Mengtow	Randomly sampled threads	Forum Thread	Random Draw	6
ORM	MGTOW	CS MGTOW	Relevant Selection	YouTube Video	Relevant	190
ORM	MGTOW	Goingyourownway	Relevant Selection	Forum Thread	Relevant	48
ORM	MGTOW	Mengtow	Relevant Selection	Forum Thread	Relevant	21
ORM	MGTOW	No Ma'am	Relevant Selection	Web Article	Relevant	11
CC	PUA	Roosh V	*The Best of Roosh: Volume One*	Book/E-Book	Community	219
CC	PUA	Michael Chief	*First Date Blueprint*	Book/E-Book	Community	30
CC	PUA	Nick Savoy	*Magic Bullets*	Book/E-Book	Community	199
CC	PUA	Alpha Game	"Top Posts" section, and "Foundations" section	Web Article	Community/Popularity	22
CC	PUA	Heartiste	*On Game*	Book/E-Book	Community	473

[3] 89 pages of web articles, and 82 minutes of YouTube videos.
[4] 42 pages of web articles, and 15 minutes of YouTube video.

CC	PUA	Mistery	*The Venusian Arts Handbook*	Book/E-Book	Academic/Community	209
CC	PUA	r/seduction	"Essential guides and beginner material"	Reddit Post	Community/Popularity	117[5]
RS	PUA	r/seduction	Randomly sampled posts, with comments	Reddit Post	Random Draw	134
RS	PUA	PUA Forum	Randomly sampled threads	Forum Thread	Random Draw	120
RS	PUA	MPUA Forum	Randomly sampled threads	Forum Thread	Random Draw	12
ORM	PUA	r/seduction	Relevant Selection	Reddit Post	Relevant	105
ORM	PUA	The Attraction Forums	Relevant Selection	Forum Thread	Relevant	6
ORM	PUA	MPUA Forum	Relevant Selection	Forum Thread	Relevant	20
ORM	PUA	Roosh V	Relevant Selection	Mixed[6]	Relevant	139
CC	Incels	Incels.is	"Must-Read Section", eleven most popular threads	Forum Thread	Popularity – by views	204
CC	Incels	Incels.wiki	All "Theory" pages from the homepage	Encyclopedia Entry	Academic/Community	213
CC	Incels	Incels.blog	"Most Popular" articles	Blog Post	Community/Popularity	24
CC	Incels	r/incels	100 most popular posts from 2016 and from 2017, without comments	Reddit Post	Popularity – by upvotes Academic/Community	64
CC	Incels	r/braincels	Two most popular posts for each month of the subreddit's history, with comments	Reddit Post	Popularity – by upvotes Academic/Community	463
CC	Incels	r/IncelsWithoutHate	15 most popular posts of all time, with comments	Reddit Post	Popularity – by upvotes	150
CC	Incels	Elliot Rodger	*My Twisted World*	Book/E-Book	Academic/Community	137
RS	Incels	r/braincels	Randomly sampled posts, with comments	Reddit Post	Random Draw	81
RS	Incels	Incels.is	Randomly sampled threads	Forum Thread	Random Draw	99
RS	Incels	r/IncelsWithoutHate	Randomly sampled posts, with comments	Reddit Post	Random Draw	90
ORM	Incels	Incels.wiki	Relevant Selection	Encyclopedia Entry	Relevant	96
ORM	Incels	Blackpillclub	Relevant Selection	Forum Thread	Relevant	48
ORM	Incels	Incels.net	Relevant Selection	Forum Thread	Relevant	18
ORM	Incels	Incels.is	Relevant Selection	Forum Thread	Relevant	91

[5] 106 of which include only reddit original posts (no comments), and 11 of which are comprised of reddit posts with their subsequent conversation.

[6] 136 pages of blog posts, 1 page of interview (web article), and 2 pages of excerpts from one his book: *The Best of Roosh: Volume 2*.

ORM	Incels	Non-cucks-united	Relevant Selection	Forum Thread	Relevant	3
ORM	Incels	r/IncelsWithoutHate	Relevant Selection	Reddit Post	Relevant	10
ORM	Incels	r/braincel	Relevant Selection	Reddit Post	Relevant	1
ORM	Incels	Incels.wiki Twitter	Relevant Selection	Tweet	Relevant	2
ORM	Incels	Incels.is Twitter	Relevant Selection	Tweet	Relevant	1
CC	MRAs	Paul Elam	*Men. Women. Relationships. Surviving the Plague of Modern Masculinity*	Book/E-Book	Community	208
CC	MRAs	Judgy Bitch	"Top Posts and Pages"	Blog Post	Community/Popularity	75
CC	MRAs	Karen Straughan	Five most popular videos	YouTube Video	Popularity – by views	264
CC	MRAs	Paul Elam, Tara Palmatier	*Say Goodbye to Crazy: How to Get Rid of His Crazy Ex and Restore Sanity to Your Life*	Book/E-Book	Community	262
CC	MRAs	r/MensRights	Content linked in the subreddit's sidebar	Web Article	Academic/Community	113
CC	MRAs	Warren Farrell	*The Myth of Male Power*	Book/E-Book	Academic/Community	331
RS	MRAs	r/MensRights	Randomly sampled posts, with comments	Reddit Post	Random Draw	277
ORM	MRAs	Angry Harry	Relevant Selection	Blog Post	Relevant	68
ORM	MRAs	A Voice For Men	Relevant Selection	Web Article	Relevant	145
ORM	MRAs	The American Gentleman	Relevant Selection	Blog Post	Relevant	4
ORM	MRAs	Roy Den Hollander	Relevant Selection	Mixed[7]	Relevant	52

Explaining the "Grounds for Inclusion" column labels

1) **Central Content Section**

[7] 9 pages of web articles, and 43 of excerpts from his online autobiography: *Stupid Frigging Fool.*

Each addition to this section was justified because the source/author was commonly referred and linked to in the manosphere (label "Community"), and/or in manosphere research (label "Academic"). When possible, most popular content was selected via some indicator on the source website itself - such as a "Top Posts" section - or some quantitative metric such as views on YouTube videos (label "Popularity").

2) **Random Sample Section** (label "Random Draw")

Material was randomly selected over a three-year schedule on websites from the CC selection which satisfied those two conditions: active at the time of the draw, and user-generated content only (*i.e.,* forums and Reddit).

3) **Other Related Material Section** (label "Relevant")

At the researchers' discretion, relevant documents encountered in the course of browsing the manosphere were added for qualitative analysis, especially when they pertained to life sciences, evolution, etc.
