# Supplementary material for: A hundred and two just-so stories: exploring the lay evolutionary hypotheses of the manosphere
Source: Evol Hum Sci. 2025 Oct 9;7:e41. doi: 10.1017/ehs.2025.10020 (PMC12645320; doi:10.1017/ehs.2025.10020)
Supplement: Bachaud et al. supplementary material [file S2513843X25100200sup001.zip › S2513843X25100200sup001/Supplementary Material S5.pdf]

Supplementary Material S5: Filled Template for Qualitative Analysis

**Document Information**
**Reference:**
**Title: First Date Blueprint**
**Author: Michael Chief**
**Date: 2014**
**Nature: Ebook**
**Length (in pages): 30**
**Group:** Pickup artists (PUAs)
**Link:** First-Date-BluePrint.pdf
**Quotes (with page number)**
**References to evolution:**
"It's the man's responsibility to bear ALL risks in the male-female dynamic. We human beings have evolved so that men are to protect women. We protect them from all physical harm as well as any and all risks. This includes the risk of getting rejected in any sexual advance. If you demonstrate that you're willing to bear all of those risks, she'll see you as more of a man and, in turn, become more attracted to you." (p.25)
**References to genes:**
**References to animals:**
**References to the brain and hormones:**
"The first thing you want to do on a date is go to a coffee shop to chat a little bit while drinking something that has either coffee or chocolate. The caffeine from coffee or chocolate will release dopamine and will contribute to the two of you being more attracted to each other." (p.12)<br>"Thanks to the mirror neurons in our brains, human beings are exceptionally empathetic. This means that whatever you feel, people around you feel some degree of the same emotion. Whatever people around you feel, you're going to feel some degree of their emotions. This is why women feel awkward and uncomfortable around you if you feel awkward and uncomfortable. On the flip side, women will feel more relaxed around you if you feel relaxed." (p.20)
**Sex differences:**
"Women can smell desperation from a mile away, and it smells really, really bad." (p.4)<br>"Women generally have a better sense of smell than men" (p.14)
**Other references to science, life sciences, rationality, etc.:**
**Sources of the scientific knowledge/information about its propagation:**
"And, if she thinks you've spent more time together with you, the more comfortable she will be around you. You'll naturally have more rapport. You'll have more trust. You'll be more familiar with each other and, as social psychology has shown, that will lead to her liking you more." (p.6)
**In-group/Out-group controversies:**
"Sparking or building or maintaining attraction can actually be a pretty simply process. Lots of "gurus" from my industry overcomplicate it with unnecessary fluff like "negging" and "DHVs." There is absolutely no need to overcomplicate attraction" (p.19)
**General Information**
**Key information about the group:**
"Before you head out, though, I'd like to tell you something else. I want you to really know that women are human beings who deserve your love and respect. There's a lot of misogynists out there who want to

dominate or hurt women. Don’t be one of those guys. Be one of the good guys who spreads love and selfless intentions. Focus on making other people feel good and karma will repay you with happiness and endless satisfaction. We have a saying in the pickup artist community that goes, “Leave her better than you found her.” (28-29)
**General manosphere information:**
**Other/Miscellaneous:**
Talks about the patriarchy while discussing LMR: “However, because of the sexual double standards borne from our patriarchal society, many women believe that they have to protect their sexual purity, even if they’re not virgins » (p.27)
**Summary/Analysis**
**Interest: Gravel**
**Summary:**
A short first date guide.
**Analysis:**
A good representative of the new school of pickup present on r/seduction, less jargon, and more careful not to appear misogynistic. Also, there are a few references to science.
**Tags:**
LMR; neurons; patriarchy; in-group controversy
**Other Potential Data**

Notes on the document

Quotes from the material are extracted and classified into broad disciplinary and thematic categories.

The relevance to the research (“Interest”) is coded from 0 to 4 (No Interest – Sand – Gravel – Stone – Rock).

Tags are used for later search, retrieval, and analyses.
