# Supplementary material for: A hundred and two just-so stories: exploring the lay evolutionary hypotheses of the manosphere
Source: Evol Hum Sci. 2025 Oct 9;7:e41. doi: 10.1017/ehs.2025.10020 (PMC12645320; doi:10.1017/ehs.2025.10020)
Supplement: Bachaud et al. supplementary material [file S2513843X25100200sup001.zip › S2513843X25100200sup001/Supplementary Material S6.pdf]

Original Just-So Stories Repository

Here is the comprehensive repository of the original manosphere material excerpts from which the JSS dataset was extracted.

1)

Women will spend hours a day, sometimes turning themselves into beautiful and sexy objects, they wear high heels because small feet correlate with high estrogen levels and therefore fertility and feet look smaller in high heels.

2)

Adjustments were made when they could be, to keep as many women as possible within the sisterhood. This is where you'll find a ton of attention in female spaces given to things like tone and being nice and emoticons with smiling faces and getting along, even when there are disagreements. A lot of their interactions are about comfort level and feelings of acceptance. Men, on the other hand, lack the hardwiring to form a preference for maleness based merely on maleness. And that really just makes sense when you think about men's roles for the last couple million years or so, roles that involve things like beating the men down the valley to a pulp when they threatened women and children, or competing against other males within his community for a shot at the mating game. Given those roles, automatically siding with one's own gender over the other is just not going to work. And it's not that men can't manifest any forms of overt preference, it's just that when one group preference manifests in males, it just isn't based on maleness alone. There has to be a common purpose, a common set of ideals, of principles, a common duty or cause, a common doing or a common position in the status hierarchy. […]

Now, this is the most common path, in my opinion, to a positive male identity, because men lack a mechanism for automatic own-group preference. Siply put, they just don't relate to other men automatically just because they're men. Women have this bias, which provides them a natural ability to form cooperatives and relate to other women and seek consensus through their strong mechanism for own-group preference based on gender alone

3)

And in much the same way that female chimps needed to develop their non-verbal manipulative skills in order to negotiate successfully with the alpha males with whom they wished to mate and to live amongst, so it is that human females have also developed greater *language* skills than males in order to achieve more or less the same ends.
[…]

But these female chimps are also playing with fire. They must be able to give off signals that protect themselves from attack. And the best way (in fact the *only* way) to do this is to appear to be as non-threatening as possible. And the weaker and the more vulnerable that they look to the alpha male, the less of a threat does the alpha male perceive them to be.

And, putting it bluntly, this is why human females are rather like young children in many respects – squeaky voices, not much body hair, round bits, soft skin and, of course, inexplicable tantrums. The idea is to look and act vulnerable – just like children.

4)

And in the past, those little societies that spent their time emotionalizing, instead of creating, inventing, and progressing, had no chance in evolutionary terms. They lost the battle

long ago. The men were killed and the women were carried away. And they no longer exist. They have been statistically washed away.

5)

When the females of a species invest significantly more energy in to gestation than males, as is the case for humans, then the size of the next generation is largely constrained by the number of fertile females. As a result of this, a species can generally tolerate the loss of a male more easily than the loss of a female. It thus makes sense for a species to allow males to vary more in their characteristics. If a characteristic is disadvantageous to a species, then the loss of males will be less damaging than the loss of females. If a characteristic is advantageous, it can be spread to the rest of the species. Thus, testing new characteristics on males is an advantage to a species overall, and so we find men vary in their characteristics more than women. In effect, the loss of women will constrain the size of the next generation while the loss of men will constrain genetic diversity. This does not justify male disposability, though. Even if men were biologically disposable in the past, they no longer need to be. The Earth carries more than 7 billion people today and could be carrying 10 or 11 billion within a few decades. We no longer need the ability to rapidly recover numbers, thus any presumed bias toward male disposability that existed in the past no longer applies.

6)

Female Islamic dress codes are cultural practices similar to foot-binding in China and female circumcision (genital mutilation) in sub-Saharan Africa and other regions, in that they are all cultural codification of female intra-sexual competition to secure high mate-value pair-bonded sexual partners. They signal to prospective male long-term partners a commitment not to engage in extra-pair sex.

Bear with me (for just one paragraph) while of necessity I explain this.

The whole point of pair-bonding is to increase the reproductive efficiency of the female by the presence of the male partner dissuading sexual access by low mate-value males. It is not about male control of female fertility as usually supposed. Researchers have been flummoxed by findings that pair-bonding does not prevent the female from choosing to have extra-pair sex. This should not have come as a surprise, because it's in the female's interests to have sex with males who have superior genes to that of her pair-bonded partner, and there is nothing much the male partner can do about it – not least because the extra-pair male would be more dominant, and likely would win any physical contest if it came down to this.

But if the male's job is to keep away lower mate-value males, then he needs to be assured that the female does not have a propensity to engage in sex with any males not too much lower inmate-value than himself; otherwise his job as 'guardian' is made much more difficult, and not worth the bargain he struck of trading off (to some extent) his own relative lack of mate-value in return for his provision of a mate-guarding service.

This is how face-body coverings, foot-binding and FGM work. They are widespread fashions among women that aside from their particular cultural manifestation are essentially a unitary biological phenomenon. As with all fashions, they are not imposed but readily adopted as women wish to join the more advantageous in-group and to dissociate themselves from the relative under-dog out-group.

It requires no imposition from without. Yes, of course, men will at the behest of women reinforce such behavior. In being at the civic end of sociality (the locus of their own intra-sexual competitiveness), men are in the position that women will appeal to them to act on their behalf to where necessary help impose uniform female cultural practice. This is not in any respect ‘oppression’ by men. If anything it is a imposition on them by women to which they feel dutybound to accede.

7)

This is all well and good, except that there is another dynamic playing out in gender differences. There is something called sexual dimorphism. This is a term from the mainstream biological sciences where the male and female evolve to acquire different phenotypic traits. And in the case of intelligence, there are actually grounds to infer that provided-for women who never have to do anything except shop shop shop, text text text and dance dance dance finish up actually losing their intellectual capabilities. In their provided-for lives, without moral responsibility, their brains actually appear to rot. They do become dumber.

8)

One among several reasons is, that women and children are permitted to complain, whereas men may not. Once again, the evolutionary sense of this taboo is to stop low-ranked males (as the clumsy adolescent still is) from getting “undue access” by complaining and begging for what sexual selection by rank denies him.

9)

Evolution also created a chemical reaction in heterosexual men’s brains that causes desire for women. The drive for sex provides a chain to tie the male to the female so that his protection of her will continue after she bears children while compassion ties him to protecting the child. Evolution is no one’s fool and neither are females no matter how much they feign naiveté. Feminine propaganda over the ages depicted men as lower-life forms driven only by sex when in reality women burn to copulate more frequently because they derive more pleasure from it. Evolution required women to enjoy sex more; otherwise, no female would risk the pain, burden and sacrifice of childbirth and rearing just to get laid if the pleasure she felt equaled that experience by a man. Women once again twisted the differences between the sexes to their duplicitous advantage by pretending they were doing a guy a favor by sleeping with him, when in reality he was doing her the favor.

10)

A woman’s drive for sex and economic support, which is the modern-day form of protection, made infidelity a way of life for her. Females spread their bets, so if one man bites the dust, either physically or economically, she still had other beaus to depend on. To keep her beaus tied to her, she needed to cheat on all of them but still convince each with her tears, entreaties and sex that he was the only one. Over millions of years, natural selection eliminated the faithful females, since they tended to die out with only one male protecting and supporting them. That left modern-day man with only a huge pool of hos—billions of them.

11)

Even with such new evidence, I still faced the Feminazi belief system that only men should receive punishment for adultery, since they are natural philanderers. Once again “new age women” got their facts wrong.

Over millions of years of evolution, natural selection favored promiscuous hominid females. Prior to ten thousand years ago, humans lived in tribes with the men providing the meat by hunting animals and women providing berries, roots, nuts and other staples from foraging. Survival required the protein from meat, which women generally could not acquire themselves because evolution provided men with greater upper body strength and the spatial acuity that facilitated hunting wild animals. Men's more athletic bodies also enabled them to provide protection to women, especially during pregnancy and afterward when both the nursing mother and newborn were highly vulnerable to the dangers of the wilds. To assure their survival and that of the species, women and men entered into long-term relationships, generally four to five years, in which the man provided for and protected the woman and her offspring. The main occupation at the time for men, hunting wild animals or fishing, carried a high risk of death. A woman quickly realized that the man on which she depended, not just for her survival but the survival of her genes through her offspring, might one day end up as an animal's lunch or swim with the fishes. In order to assure a continual supply of protein and protection, many women simply used the currency of their bodies—sex, to make "special" or "good" friends with other men. Naturally, these women tried, with varying degrees of success, to hide their infidelity from their respective main man, but over time, men, initially at the disadvantage by willing to give someone the benefit of the doubt, eventually realized the slut-like nature of their trusted confidants. Men, just like women, wanted their genes to survive through their offspring, but unlike women, a man could not know for certain whether an offspring was his due to the promiscuity of the mother. So to compensate, men engage in sex with other women figuring one of them will bear a child with his genes. As the millennia passed, the genes of the more successful tramps survived while those of faithful women went extinct, which left us today with a whole lot of hos.

12)

One of the many reasons evolution made men better suited to handle the stress of social power was to curtail the boundless evil that females drunk with power will pursue. Witness those high school sororities that were hazing new recruits in a field out in the West. I went through hazing at a college fraternity that was nowhere near as brutal as what those girls were dishing out. And all that it would have taken to stop those barbarian princesses was for one man, just one and not an androgyny, to walk into that field and tell them to knock it off. Or take the female customer service reps that the public has to deal with when a company makes a mistake. Reasoning with them is impossible. They lie to cover up their ignorance, can't make a judgment call to solve the problem and use authority in an arbitrarily vindictive manner. Men are not immune from abusing power, but because evolution slotted men for handling the responsibility of social authority, they are more able to handle it and less likely to abuse it.

13)

After going to the Bolshoi Ballet in Moscow plenty of times, I concluded girls are just better dancers. Probably has something to do with displaying their wares around the primeval campfires or they just have more with which to work.

14) 15)

Females handled the kids in the early years that resulted in women developing superior aptitudes in language that grew out of understanding baby gibberish and the ability to sense emotions, which together enables them to read the signs of problems or normalcy in a child. While caring for children, women gathered berries and roots that developed their manual

dexterity in which to this day the average girl excels over the average man. Watching the kids and searching for small items of food at the same time created in females a speed of eye movement generally quicker than in men. For example, it's difficult for the average guy to catch the average girl looking at him because her eyes move more rapidly than his. The rapid eye movement ability in females makes for excellent accountants and secretaries.

16)

Until late adolescence, females have the advantage over males because they mature faster, which masks the underlying difference in IQ. Men have larger brains even when you take into account their larger body size. That means there are more neurons, which probably gives guys an advantage in processing information. The difference may date back to the Stone Age, when females sought out men who were more intelligent than them in a bid to pass on the best genes to their children.

17) – Answering a Reddit post entitled "What is the evolutionary benefit for a woman to be slutty."

There is no benefit.

One of the fundamental ideas underlying modern seduction is this: our instincts are out of place in the modern world.

Our brains are designed to live in a stone-age tribe of about 150 people, give or take. In that situation, each female's job is to secure commitment from the highest-value male she can. Her tool for securing that commitment is sex.

In a small tribe, everyone knows everyone else, and they also know *each person's status.* The tribal chief is highest status. There's the second-highest status guy, his son. And then there's the 3rd, 4th, and 5th, etc.

If the chief's son picks a female to mate with, she will *instantly* go fuck him, no game required. Why? Because this is her best chance to garner resources for herself and her offspring. In fucking him, she hopes that the oxytocin and assorted bonding chemicals will take their effect on him, and cause him to desire commitment with her. In a small tribe, this was likely to result in a pair-bond, due to the simple fact that *there weren't a lot of options.*

In the modern world, we live in cities with potentially millions of strangers. There is no "chief." So, there is NO LONGER SUCH A THING as "absolute status." "Status" is now entirely based on social skills, aka Game.

When a woman goes into the modern-day nightclub, there could be five or ten guys who have the requisite game to attract her. Her instinct, again, is to fuck the high-status guy ASAP, because that might be her only chance to exert that sweet cocktail of bonding chemicals.

Unfortunately for sluts, they are no longer in short supply. In a tribe of 150, you can't pump-and-dump a woman; there might only be twenty-five females of eligible age. In a modern city, a Player with Game can run through hundreds of women without any negative repercussions.

Sluts get screwed over because they are indulging obsolete instincts. It's just like our desire for sugar: back in the day, it was only found in fruit, which is generally a healthy thing

for humans to eat. Nowadays, we have refined sugar everywhere; and legions of people get obese and develop diabetes.

Sluts are like the obese diabetics of the sexual world: they are over-indulging in instincts that were once beneficial, but now are harmful.

18)

Surprisingly few men know how to flirt. (It's surprising because, given the importance of flirting to evoking a feeling of incipient sexual release in a girl's mind, you'd think evolution would have ensured a lot more men are skilled at the craft. I consider the absence of widely distributed flirting skills, particularly among northern Europeans and Asians, to be evidence that for much of mankind's ancestral past the sex ratio was skewed enough in the typical man's favor that he didn't need to learn how to appeal to women's romantic needs.)

19)

[ed: women are the reproductively more valuable sex, and so it makes sense that evolution would have "gifted" women with an oversized entitlement complex and the inability to engage in self-criticism.]

20)

Jettison politics from your personal life. Jawing about political ideology is worse than useless — it's a time suck and a trick played by your status- seeking reptilian hindbrain on your frontal lobes that does nothing to bring you more happiness OR status. Your vote really won't matter. Don't believe me? When was the last time a significant election was decided by one vote?

21)

Women are designed by nature to begin the next generation not much older than age 25. Her risk of miscarriage or fetal abnormalities increases each year after that and exponentially so after 35. Her body begins to wear down which affects how much energy she can devote to raising small children. If she has not found a suitable mate by her late 20s she will begin to notice that those powerful feelings of infatuation she felt for crushes when she was younger, perfectly created by evolution to bring a man and woman together to make babies, now seem muted and foggy.

22)

What is missing from this article is the opportunistic and fickle nature of women…think into it logically… when it suited them to rely on men for resources because the world was a tough and nasty place, they hid at home and 'took care of the kids'… while men got killed fending off the wolves (and other men)…. Most species share the load equally or in fact leave the females to take care of the kids on their own….. now it's relatively speaking much safer and easier…they don't need to hide any more… they want a piece of the action… a piece of the action that men built…..

23)

It's the man's responsibility to bear ALL risks in the male-female dynamic. We human beings have evolved so that men are to protect women. We protect them from all physical harm as well as any and all risks. This includes the risk of getting rejected in any sexual advance. If you demonstrate that you're willing to bear all of those risks, she'll see you as more of a man and, in turn, become more attracted to you.

24)

Let's take relaxed shoulders as an example, which is just one of dozens of elements of confident body language. There's nothing inherently attractive about relaxed shoulders. Look at yourself in the mirror. Relax your shoulders and let them fall back. Now tighten them and raise them. Do you think you look any sexier or less sexy? Probably not. However, confident men are disproportionately likely to spend more time with their shoulders relaxed. Because they are confident, they can do this. They don't have to be on edge all the time, always vigilant for threats. They are secure in their position and their skills. (They'd better be, or their confidence will be their downfall…) Over time, women who like men with relaxed shoulders tend to end up with a greater share of confident men. And if confidence is correlated with success, then this means that they end up with a greater share of successful men. That means their children will be more successful. Over time, their children will overwhelm the children of women who mate with men with tense shoulders. Even though there's nothing inherently sexy about relaxed shoulders, or eye contact, or standing with your feet a decent distance apart, or any physical indicator of confidence, these little things add up to make all the difference in the world

25)

Our switches are wired to respond to qualities such as a specific hip to waist ratio, breast shape and size, facial and body symmetry, and other youth and health indicators.

If a woman possesses and can demonstrate a certain **quality** that one of your sexual selection switches is programmed (evolutionarily adapted) to respond to, you'll feel immediate attraction for her, without the need to even think about it. In fact, **you** won't even have **a choice** about it.

It is interesting to note that…

- 80% of a man's attraction switches are set to respond to a woman's replication value. Only 20% respond to her **survival** value.
- Only 20% of **a** woman's switches **are** set to respond to a man's replication value, while 80% respond to his **survival** value.

26)

Woman are really good at figuring out if a woman is good or bad for her son. Remember that a primary objective of a woman's DNA, tuned by millions of years of evolution, is making sure her sons pass on as much of her DNA as they can.

There are edge cases where that's not true (like this story) but I bet there is more to the story.

27)

If you get addicted to bad behaviours, laziness, don't work on yourself, and get depressed, this is a *signal*. When you try to get girls into your life, they *sense* it and you get feedback on your worth.

Women can't help but to reject you if you're in this haze. It's in their biological code. It's a blessing in disguise and an indicator that there is something you're doing wrong that needs to be fixed internally.

It's really interestinf seeing how this works. I think this was an evolutionary development that kept men on their best behavior for the sake of our survival. Maybe women lost attraction for men that got too lazy and became a risk for their safety as well as the tribe's.

28)

Sex also represents a much larger investment for a woman than it does for a man. Her reproductive years are fewer than his and her time spent producing each child is much greater as well — ten minutes of sex for him, nine months of pregnancy for her. The result of this is that women are considerably choosier than men, and it is also much more important to a woman that a pair bond exists before she will engage in sex. For this reason, due to natural selection, women often experience anxiety just before having sex the first time with a new lover. It's not her fault — this last-minute resistance is a pre-programmed emotional response. Her emotions are only trying to do what's best for her. (This one sounds like classic David Buss: did we miss it?)

29)

Rejection and Approach Anxiety Logically, rejection causes us no harm. But emotionally, rejection can be a punishing experience. To understand this, we must look at the ancient environment for which we were designed. In a tribal group, there will be some small number of available women of breeding age. When a man approaches one, he risks rejection, and if that happens, all the other women will know, which will diminish his value in their eyes — maybe to the point where none of the women will mate with him. This is called preselection — women look for social validation of their choices. A suitor who is preselected will be more attractive, whereas a man who has been rejected will be less so. Another factor regarding approach anxiety is the possibility that she may already be taken, in which case there is a component of real, physical danger to any male who approaches her. For all these reasons and more, men are naturally selected to experience approach anxiety. Logically, of course, modern society fixes these problems. If I am rejected, I can simply go to another part of the bar, or leave the bar entirely. I will probably never see any of those people again. But my emotions don't know that. My emotions are trying to do what's best for me.

[…] Men take a larger risk than women when first approaching. In ancient times, this posed a legitimate safety concern and thus men still experience approach anxiety.

30)

Having not been a caveman for some time, I can't really speak to what their world was truly like, but I imagine that the world of the caveman is composed of things that are basically harmless, and things that are totally life threatening.

A squirrel? Harmless.

A grizzly bear? Will kill you.

Sunny day? Harmless.

Cold rainy day? Pneumonia will kill you.

Fellow tribesmen. Probably harmless. But, if not harmless, will kill you.

Man from another tribe? Will probably kill you.

Coyote? Won't kill you, but it'll ear your babies.

Compare that to our modern world. There are few things that are life threatening, but lots of things that suck in a moderate degree. Car wreck? You'll probably survive, but maybe with some broken bones, and you have to get a new car, deal with the insurance, lose some money, etc. Test at school? It only counts for 20% of your grade for the semester, which is a fraction of your overall GPA. Caveman didn't have "20% of your grade for the semester." Fail a test as a caveman, and you bleed out and die.

My theory is that our brains are wired to feel either unafraid of something, or super afraid of it, and aren't really well adapted for feeling a moderate amount of fear. Apply this to approaching, and our brain has to make a choice. Do you feel totally zen, or do you freak out? Your brain knows there is some risk to approaching – rejection hurts, you might be embarrassed, she could have an overprotective boyfriend, your friends might see you strike out. While all of these things are only small risks, your brain has no choice but to treat them like a hungry grizzly bear. Thus you feel a level of anxiety that is completely out of proportion to the actual risk you're taking. That is approach anxiety.

31)

Did you also know that the fear of rejection may in fact be an evolutionary instinct that kept us alive? Back in prehistoric times a person being social rejected and cast out on their own by their clan meant that you'd probably die alone in the wild. Those of us alive today are all descended from the people who managed to not be outcasts, or the outcasts who were able to find a clan that liked them enough to take them in. Those with a stronger fear of rejection were less likely to behave in such a way that would cause their clan to cast them out, and if that's true then that explains why the fear of rejection is so prevalent.

32)

Human beings have basically 2 main desires: to be at the top of the hierarchy (or as high up as they can get) and, if they can't get to the top, to be accepted by the people at the top. I think a large part of approach anxiety is the fear of getting rejected by the tribe. It's not a logical fear based in reality (i.e., who cares if this one girl doesn't want to talk to you?), but rather it is something primal rooted in our DNA. That's why you can't defeat AA with logic – no matter what logical thoughts you have, your emotions will create more excuses in your head as to why you shouldn't approach. That said, I think women have much stronger rejection anxiety that men do, which is why society has evolved such that men always have to approach.

33)

Women have evolved to be perceptive of a man's emotional state and body language is the physical manifestation of inner game, so that's what women key in on first.

34)

I read a great deal of dating advice, most of it bad. This includes dozens of blogs, countless women's websites, even the lady magazines like Cosmopolitan. I also talk to every woman I can about dating and relationships. Yes, I know, talk is cheap. When I discuss these issues I'm trying to read between the lines and look for descriptions of actions, not words from

the rationalization hamster or aspirational lying. There is one particular and common theme from female dating advice-givers that has perplexed me:

Men want a challenge.

The usual female explanation for this theme is that "men are hunters". That much is true. Yet when our paleo ancestors went hunting, it was for food. It was for survival. If the men didn't bring home the animal protein, the tribe withered and died. After spending a few incredibly tough days trying to slay an uncooperative animal, why would a man return to the clan and have to hunt all over again for a compliant female willing to mate? It makes no sense. From an evolutionary psychology point of view, it's completely counter intuitive.

It took some thought to figure this out. I'm slow, but I eventually get there. Here's the basic set up:

Women seek the top 20% of men. The women must hunt these men because these men have options and will only select a small percentage of women to whom they will provide DNA and possibly resources. This makes perfect sense. Top paleo providers were in short supply. To hunt and land one of these men, even if for a harem arrangement, was a successful survival strategy for the woman and her offspring.

Let's fast forward to our modern, industrial culture. Armed with the power of projection and a healthy rationalization hamster, women think because they must pursue men (the top 20%) that men must pursue them. It goes along with the modern projection that because women are attracted to masculine accomplishments, that men must also be attracted to masculine accomplishments in women.

It gets worse. The guys not in the top 20% must do the pursuing of the top 20% of women (attractive and feminine women, not the masculinized career dames). The women's projection that men must give courtship chase is utterly reinforced by an army of guys without Charisma trying desperately to win these women's romantic (um, sexual) favor.

Men with Charisma do not want to hunt women. They want compliant (PC word alert!), feminine women who easily recognize the man's achievements without drama, bitchiness, and demands. Sure, Charisma is required because our recent industrial age requires women to be a bit coquettish. This is the price for not having to worry about a saber tooth tiger eating the women and children while the man is out hunting mastodons.

A woman wants a man without Charisma to hunt them because this is a validation of her desirability, nothing more. The woman doesn't want the DNA (sex) from that man, she wants the emotional support in addition to the desirability validation. This is manifested in contemporary times as the FriendZone and weaker men being the emotional tampon.

So let's summarize – Women want beta men to hunt them. They want to hunt Alpha men.

35)

Race mixing (you seem fixated on Bantus, but it's more than that) has benefits – such as the averaging out of features, which leads to more attractive offspring. If your previous 20 generations are relatively endogamous and you crossbreed with someone less genetically close to you, your offspring will have more average phenotypes (= more attractive).

This is why the most attractive region of the world is the former-Roman areas – the mediterranean. There are among the most mixed region because they were crossroads for empires and commerce.

36)

The simple fact of the matter, however, wasn't that the men were trying to be sexist. They were merely competing . . . *among themselves*. Even when they were faced with a competitive female co-worker, and one who could easily challenge them at their work, she wasn't included in the competition because *when men compete it is a matter of sexual selection, not merely a desire to "be best."*

That's one reason competitive endeavors are so often the target of feminists. When men compete with other men, they don't like to include women. Even when you do, they'll find a way around it. So for feminism competition is a negative, as it hurts women even if it's absolutely vital for the healthy sexuality of the men.

Women often mistake this competitive tendency for blatant sexism. The fact is that we consider measuring ourselves against women to be pointless – we aren't *competing* with women to display our high sex value, we're trying to *attract* women with our high sex value, and confusing the two does us both a disservice.

37)

The instinct to pair-off eligible menfolk to "quality" single women they know is almost irresistible to most women. It's not a moral failing, as some would see it, it's a *product of their biology*, as the esteemed **Athol Kay** terms it, their "Body Agenda": pairing you off with a lesser female reduces competition in the SMP, improves her position in the Female Social Matrix, and gives her a smug feeling of satisfaction that she has "brought love into the world" by her matchmaking. It's not a moral failing . . . *but that doesn't mean you have to play*. Or *should*. Consider it an institutionalized biological Shit Test .

38)

One of the bigger problems plaguing today's woman is that she's lost touch with one of her most powerful evolutionary advantages—a well-calibrated internal clock. *Remnants* of this ancient gift are still visible, if you look closely. On average, I'd say, women are more consistently punctual and meet deadlines more often than men. In college, it was always the girls who left the library the earliest, having finished their work for the day. Past, say, 10 pm, the 24-hour library on campus always became a brutal sausage fest. Say what you will about the *quality* of the work, the female gender—everything else being equal—is better at getting shit done on time.

For much of human history, this monitoring of time included a keen awareness of the time-limited nature of their fertility and their *most* valuable evolutionary commodity—their beauty. A properly raised girl used her prime years to lock down a quality man who, enamored with her youthful charms—and the prospect of enjoying them for several more years—signed up for the long-term membership. Women made sacrifices, but understood that the alternative was also a sacrifice.

39) – THREE OCCURENCES

Women are Machiavellian by nature. In comparison to the average man, they are far more proficient in matters of persuasion and general social manipulation. The theoretical evolutionary basis for this sex difference is that due to smaller body mass and inferior musculature, women evolved to attract and use men as tools rather than directly compete with them.

Women are machiavellian in nature, this means they are comparatively proficient at being manipulative versus the typical male. The evolutionary theoretical basis for this is due to smaller size and inferior musculature women had to learn to use men as tools rather than directly oppose them in a physical conflict (as they would undoubtedly lose in all but very few scenarios) this makes the pronunciation of their strength a propensity to be mentally rather than physically violent.

Women are naturally more socially/emotionally perceptive than men can even comprehend. Evolutionarily, as the physically inferior sex, woman have relied on cunning to ensure their survival for thousands of years.

40)

Masculine energy is all about breaking through barriers, overcoming obstacles, and achieving goals. Evolution has made us this way, of course. Men are logical, and approach problems with a framework based on logical assumptions with a purpose of overcoming some barrier.

But women aren't like this. It's a mistake for men to project onto women our own thinking patterns. Feminine energy is all about bonding, finding emotional connections, and opening up to receive love. They are not guided by strict logic. They operate more on emotional and intuitive bases. Nurturing is not breaking through barriers or crushing obstacles.

41)

Now in man, as in all animals, a peculiarity of reproduction-and-nurture is this: that of all the major tasks of the species, it is the one for which the innate programme is most complete, and the one, therefore, which requires the least intellectual effort for its performance. In plain English: a woman does not need to use her brains to have a baby, and doesn't even need to use them much in order to see the infant through the period of its most extreme helplessness. Nearly everything she needs to know is already written in her inner manual.

One of the facts about Darwinian evolution, and one so clear that it was noticed long before Darwin investigated its cause, is the fact of specialization. Animals that are fast tend not to be strong; animals that are strong tend not to be fast. An animal that might be both strong and fast will in fact be neither. Nature is very stingy when it dishes out resources and abilities. If there's no need to fly, an animal will not fly. A woman does not need intellectual capacity to have babies. Reproduction uses a lot of energy and resources but those resources are not intellectual. Men are free of the drain on resources that is reproduction, they, however, have other functions.

The intellectual activity which is required for successful hunting is extremely great… One would expect that the sex not burdened with reproduction-and-nurture would shoulder the main burden of those other major tasks which are intellectually more demanding; and therefore, by the principle of parsimony, that men will have a higher degree than women of what is peculiarly required for those tasks, intellectual capacity. I do not claim that this inference is inevitable, but it is at least a natural one. And vague as its premises are, they do furnish, I believe, the lines along which an explanation must be sought for the intellectual difference observed between men and women.

42)

You can attribute whatever legitimacy you want to studies like this, but the evidence points to higher testosterone levels as playing an influential part in sexual attraction. Also bear in mind that pheromones influence women living in close proximity to each other to synchronize their menstrual cycles – another evolutionary mechanism believed to ensure fertility and communal support for social animals.

43)

From a biomechanical perspective, the indication is that men who consistently masturbate are essentially broadcasting their status as Pheromonal Betas – and women's biochemical mechanics subconsciously registers this about them. Higher testosterone males manifest their sexual viability in both sexual assertiveness and scent. If you are chronically depleted of testosterone, and/or subjected to the calming effects of oxytocin your sexual viability is at a disadvantage. In fact, from an evolutionary standpoint, the Beta males of our feral hunter-gatherer beginnings would be more prone to masturbation as a sexual release since, theoretically, they would've had less access to breeding opportunities than Alpha males. It would then follow that definitive, subconscious behavioral and chemical cues would evolve to aid females in selecting the best mate for parental investment.

And now for the disclaimer; I'm not a endocrinologist, biochemist or physician. I'll admit this is a work in conjecture, but it's plausible conjecture. For the record, it's not about 'less' desirable pheromones, it's about a lower incidence of any sex-cue pheromones due to depletion and the behaviors that depletion prompts. It stands to reason that women would be more attracted to men motivated to being sexual with them, manifesting this in chemistry and behavior, than sexually unmotivated men manifesting signs of disinterest.

44)

All of this kind of brought me around to thinking about the psychological 'software' that's been evolved into our species as a result of environmental adaptations of the past. In War Brides I go into detail about the Stockholm Syndrome women seem to have an inborn propensity for, which logically makes them predisposed to abandoning emotional investments more readily than men. Considering the brutality of our feral past, evolving a capacity for quick emotional abandonment and reinvestment would've been a valuable survival trait for women (thus insuring a perpetuation of the species), however, in the present it serves to complicate newly developed social dynamics in terms of parental and ethical considerations

45) FIVE OCCURENCES (SHIT TESTS)

On the contrary, women evolved the instinct to push a man's buttons as a way of testing his willingness to face conflict head-on (what we call shit-testing). A man who is willing to fight against her will also fight FOR her. Likewise, a man who caves before her will most

certainly cave before his enemies. This is why bossing her around is key. She is evolved to push the boundary by picking fights with you. So unless you are strict with your girl, she will become as selfish and insufferable as you let her get away with.

This is why women have psychologically evolved a subconscious propensity to shit test; to verify the legitimacy of a man's frame.

[…]

Women's shit testing is a psychologically evolved, hard-wired survival mechanism.

This is where the evolutionary theory comes into play: you're demonstrating her faux negativity doesn't phase you and that you're an emotionally developed person who isn't going to melt down at the first sign of trouble. Ergo you'll be able to protect her when threats to her safety emerge.

A girl will purposefully (and often times subconciously) test a mans strength by creating tension to see how he responds to it. A man who cannot handle tension with a 115 pound girl probably can't handle it with a 300 pound man, so by seeing how he reacts to these situations she can test whether or not he would have been an "alpha" in the group thousands of years ago. These tension creations are commonly referred to as "Shit tests."

I believe its a hard wired evolutionary trait for women to instinctually pick fights with men. From the evolutionary research, basically women from prehistoric times had to constantly test the health and fitness of men near her. Hence the storied "shit test", and why women instinctually pick and escalate verbal fights .No matter how high value the guy is, she always has to start petty shit to make sure he's still Top Male.

If he rises to the challenge so much happier she gets. If he actually physically responds, then sell never leave him because she knows if he'll fight her despite being a couple, he'll fight anyone who threatens her and said offspring.
Since all women seem to possess that instinct, it's all the more noticeable among the counseling profession-its a socially sanctioned opportunity to verbally challenge guys , and he can't do a thing about it. – (this one is MGTOW)

46)

The root of this is grounded in women's constant, in-born psychological quest for security. Hypergamy, by necessity, makes for solipsistic women in order to best preserve the survival integrity of the species. That's not to say women can't sublimate that impulse as necessity dictates, but just as men must sublimate their sexual imperative, women begin at a point of tempering the insecurity that results from hypergamy.

47)

The purpose is fighting the female instinct to submit. Female humans have a powerful built-in instinct to submit to the commands of dominant males. It is involuntary. The instinct itself is a reflex born of thousands of years of tribal warfare, when power constantly shifted between

combating males and their tribes. Those women who were strong-willed, defiant, and loyal to their abstract tribal affiliations were slaughtered or brought to heel. Those women who were arbitrary, suggestible, and obedient to their new conquerors were safely absorbed into the population of the invading tribe and continued to breed. In either case, obedience was promoted, and defiance was selected out.

48)

Remember, women are children: mentally, behaviourally, evolutionarily. They are not like us. They don't think like us, or have the same deep sense of personal responsibility. Even the most sociopathic man will intuitively know when he has crossed a boundary and offended another man. Whether or not he feels guilty about it is a different issue, but he at least knows he's done something wrong. Evolving this instinct was the key to a man's ability to either strategically make enemies or avoid unwanted conflicts. Women, on the other hand, evolved no such instinct.

49)

As the leader, the alpha male makes all the rules for the tribe, which means that he essentially creates the rules for reality because humans did not evolve to understand the difference between objective reality and the alpha's rules.

50)

The archetypal alpha male

Betas did not evolve to follow and obey just any alpha male, but rather the archetypal alpha male, the **perfect** alpha male. The archetypal alpha male can defeat any challenge, has complete abundance, is completely superior, and leads in every respect. He is infinitely alpha across all categories. The archetypal alpha is a fake character created by evolution that only exists in our emotions, and is designed to drive betas towards the man that can best protect and lead them. A beta who seeks a man who can defeat some challenges is less likely to survive in the jungle than a beta who seeks the man who can defeat all challenges.

51)

The most dangerous aspect of man's desire to be the archetypal alpha is his infinite desire to fuck all women. Most men are losers that can rarely get laid by anybody, but all men have this insane desire embedded into their genetics, and once this desire awakens their life can quickly go off the rails.

52)

To some degree the media-entertainment complex is correct, but I believe that much of human behavior can be explained by deep, subconscious evolutionary wiring related to our need and desire to view the world as a dominance hierarchy with an alpha male at the top. Not only do we subconsciously view all of our relationships through the lens of this dominance hierarchy, but our subconscious perceptions of ourselves and others cause us to "act out" this dominance hierarchy in ways that we may not even consciously realize. Scientists have clearly observed these dominance hierarchies in apes, but to "prove" that they exist in humans would require thousands of experiments, most of which would be highly unethical, politically incorrect, and probably impossible. Therefore, all we have to guide is the little science we have and our own observations and experiences.

Each person is unique and almost infinitely complicated, but we share certain emotions formed through millennia of evolution, and the dominance hierarchy emotions are the strongest of these. Because the dominance hierarchy ensured the survival of our ancestors against their enemies in the jungle, these emotions are relentless, constant, uncompromising, and extremely powerful. They overpower all of our other emotions, even important ones like love and compassion, and even hijack our rational thoughts. When repressed or ignored, these emotions reappear in a different form, oftentimes even stronger, and sometimes disguised as something else.

53) THREE OCCURENCES (MALE DISPOSABILITY)

Humans like ourselves have been around for about 100,000 years, and earlier hominids similar to us for another 1-3 million years before that. For the first 99.99% of humanoid existence, the primary purpose of our species was the same as that of every other species that ever existed - to reproduce. Females are the scarcer reproductive resource, since the number of babies that can be produced does not fall even if most men die, but it does fall for each woman that dies (humans did not live much past age 40-45 in the past, as mentioned earlier). For this reason, the human brain continued the evolutionary hardwiring of our ancestors, placing female well-being at a premium while males remain expendable. Since funneling any and all resources to women closely correlated with the survival of children, both men and women evolved to see this status quo as normal. The Female Imperative (FI) was the human imperative.

Yet, our hardwired brains have not adapted to this very recent transformation, and perhaps cannot adapt. Women are programmed to extract resources endlessly, and most men are programmed to oblige. For this once-valid but now obsolete biological reason

----

This dates back to our hominid ancestry. It's all about the reproductive output of your tribe. Protect the females so our tribe grows stronger. Bigger tribe means more success at foraging food and being victorious in beating out rival tribes.
It's even a major tenet in the Abrahamic religions. "Have many children for the glory of God." Why do they say that? Not because it's beautiful to have babies but because you need numbers to beat out those heathens and infidels.
If your tribe consists of 50 females and 50 males, the maximum neonates you can have next year is 50. So if you go to war and lose 25 males, you can still have 50 babies next year. But if you lose 25 females, you can only have 25 babies next year. Then your rival tribe can conquer you. So it's protect the women at all costs. This shouldn't come as a surprise to any of you.

Men evolved their outgroup preference (i.e. for foids) to motivate them to protect foids in their tribe/extended family from the men of other tribes. It's extremely maladaptive in modern environment where foids are empowered to shamelessly take advantage of it. (incel rendition of this one).

54)

The feminine imperative is also why so much of what we discuss here rubs people the wrong way. Humans have same-group preference for many things, but when it comes to gender all bets are off. What scientists have found time and time again is that women have same-group preference, but men do not. Furthermore, a large portion of men in fact prefer women's group preference over men's when the two come into contention. There's some interesting theories

why that's the case, namely that those who kowtowed the feminine imperative line had better odds of reproducing

55)

My theory that I'd love to make a post on at some point is that a child's greatest fear is abandonment/rejection from the parents since death is essentially guaranteed from this in the jungle. When a random woman rejects a male to the point where he becomes depressed, anxious, etc it's because that male is because he subconsciously views that woman as his mother in a sense. That fear is a child survival mechanism projecting onto said woman. In other words betas are under developed men akin to boys.

56)

This is also why men have a physiological response in fear of approaching girls: if only the alpha male has mating access rights, and behavior detrimental to your place in the group meant death, then guys who do not feel like an alpha male will feel uncomfortable expressing their sexuality around women.

57)

3.) Being a pussy: this is an obvious one, but I mean it. By not pulling the trigger when its time to make a move, or asking for the date when a conversation has reached that point. Fear of forwardness -> implies low status.
Like asking a girl to kiss, or asking if you can touch her boobs in bed.
Intended communication behind action: I respect you as a woman and value feminism and consent
Programmed expected response: She will be more attracted to me knowing that I'm respectful and ideologically align with feminism
Reality: Afraid of rejection, has no experience, lack of social proof and dominance lead her to infer you are low on the totem pole, dont feel deserving of love in your masculine aura so you're resorting to seeking love from your feminine aura, and therefor she thinks you have bad genes and doesn't wanna fuck you.

58)

I'll expound further on this. Rejection in the wilderness amongst a protective tribe is death. When a person fears rejection (or anything for that matter) it's linked to the primal fear of death.

Exclusion from the group likely means death - This is why children have a physiological reaction to being bullied or unliked. Modern man is obsessed with reputation and how they appear to other people, because their ancestors lives depended on it.

59)

See this a lot. They don't really pay attention to where they're going but hope for the best. Walking on to the wrong elevator, etc—a function of a scatterbrained nature (passed of as "multi-tasking") but when it goes wrong, it really goes wrong. You can tell the *focus* needed to survive in the wild didn't affect them; that they avoided that kind of evolutionary selection pressure.

60)

And actually you brought up the topic of the burqa a little earlier on. And I just find that such a fascinating topic, because for sure, on one side, it's it's, as you said, the, you know, possibly the the Middle Eastern equivalent of, let's say, the wedding ring. And it allows the man to to signify, you know, kind of quasi-ownership of the female. But I guess also on the on the female side, because, you know, we're hardwired to be very, very visual. So we look for markers of genetic fitness, whether that's youth, whether that's, you know, a certain hip to waist ratio or something or a certain bust size, you know, so the burqa acts in a way to increase the value of women, because I believe in the cultures where the burqa is used, that the men aren't allowed to see the women outside of the burqa until they're fully married. And now it's the wedding night. So actually, you can't then as a man, go through the usual kind of, I guess, possibly the inspection of women. OK, you know what's her bust size like, does she have bright red lips? You know, all of the kind of markers of of genetic fitness that men are very hardwired to use. So that's a bit of an interesting one for the guy. So it's I guess it benefits the females as well as benefits the men in the particular dynamic there as well.

61)

You know, we evolved to be strong and to be able to take care of ourselves and gather meat and things like that. Right. It’s been said a million times, but it’s true. We had to get food. Right. Women, their job was to get the man. To do the food for them so they could sit in the cave and raise the babies, right. So they’re very, very strong emotionally and they can tear us apart emotionally. Your girlfriend can tear you apart. If you start telling her these ideas, she can rip you to shreds.

62)

Gentlemen,

See that unhinged twat in Seat 3A?

Shes likely to outbreed you. She will certainly outbreed me. As will Queen of Serta above. One crucial role of civilized organization of marriage is ensuring the best and the birhgtest of society procreate and thus beget productive members of the next generation.

When females determine reproductive choice, that decidedly doesnt happen. The best and the brightest are deliberately deleted from the next generation, as evolutionary law destines women to seek the seed of the most animalistic men. The rational men who do attract women realize in a hurry it’s a fools errand similarly withdraw themselves.

That means generational collapse, as every group of children are raised by entitled animal women to be entitled animals themselves. Like a bad DNA code, decay copies itself into every generation with greater frequency. If you think the current state is bad , just wait a decade or so.

63)

I think the most general imperative is, "improve your emotional well-being". I think this can result in some false beliefs. The emotions want to be satisfied for their own sake, they don't realize that they are designed to get results in the real world. So it is sometimes possible to change your perception of reality to improve your well-being, rather than to improve your material condition in order to satisfy the evolutionary purpose of the emotion.

64)

“It's because of tens-of-thousands of years of evolution. Women have evolved to become masters of manipulation. Getting the alpha-male to fuck them is as easy as "Hiya stranger!" It's getting the beta bitch to pay for his/her whorespawn for 25 years. That's the trick.... tricking him into thinking it's HIS KID is the common end game. Then there is a special kind of female spider. This one who is so manipulative she can get him to knowingly raise and pay for another man's kid(s). »

65)

We have to remember that women don’t view life the way we men do. Let’s go back to the core evolutionary purpose of a woman: to beget more kids.

Of what use would it serve to naturally select women who preferred to be alone? Their entire existence is to gestate more human beings, an act which cannot be accomplished by herself.Aloneness thus has a totally different meaning for a woman then it does a man.

A woman alone is unable to reproduce, unable to acquire assets, and unable to fulfill her reproductive mandate.

A man alone can do whatever he likes, by contrast. As such, when a man says he’d rather be alone, to a female he may as well have said he’d rather choose to be a eunuch. For a girl the state of being alone is a state to avoid at all costs.

66)

And you have a phase when she has kids. And I think this is more subtle, maybe it's less sudden, but I think once she has kids, you sort of go down further down in her list of priorities just because she cares about survival, right, survival of human race. So she doesn't care about you as much, especially as the kids grow older, I think your part is a little bit done. Um, and it sounds really sad. And I don't mean that anybody's bad, but that's kind of sad. That's something that I've been thinking about then. Yeah.

67)

Further, even a woman's mind has evolved to make her more suitable for rearing children, thus a woman's "multi-tasking brain" is more suitable to care for children - or to do other tasks while also caring for children

68)

Women have mentally "evolved" to be something intermediate between the child and the man. We have all heard that women are more emotional than men and are more "in tune with feelings" than men. And this is correct, for tell me, how do infants communicate except through the language of emotions and feelings? How does a baby indicate it needs to be fed? It is through

the emotional response of crying - certainly not by saying, "Hey momma, bring those soft, round milk thingies over to my mouth." Children communicate emotionally, and since women have evolved to become "better carers of children," they have also evolved to be more in tune to the language of children, which is emotion.

69)

No matter what men set out to do, aside from raising children, women cannot compete with men on a level playing field because we have evolved to best perform our tasks in order to make ourselves useful to women

70)

In an evolutionary survival scenario, it makes sense too, that men would love women deeper than women would "love" men. It is a man's deep love that will make him sacrifice his produced goods and even his life for the woman he loves. This rarely happens the other way around. While there is an evolutionary survival advantage for the female to lose interest in a man after completing her four year rotating polyandry cycle, there is no similar advantage for the man to lose interest. In fact, just the opposite. It is in both her and her offspring's advantage to have the man still hopelessly in love with her, providing and protecting his brains out while she slyly seeks out her next suitable mate before giving the present "tool" his walking papers. Everything a man does in a relationship is in her benefit. This is the basis of Briffault's law.

71)

Also to note, the female's position is naturally on the deceptive side while the male is trying to be as "true" as possible. There is no point in him feigning he doesn't have power like the female does. He is competing to be "the best" and if you are the best, there is no point in pretending you aren't from his point of view. It would be counter-productive to be anything less than your best.

72)

It sounds extremely blunt, but we are simply better at most things. Because we evolved to do the tasks while the women spent much of their prime years pregnant and therefore unable to do much. But, partly because of this, much more is expected of us.

73)

I have just red an article that male children inherit their intellect/cognitive genes from mothers not from fathers , they only get sex , aggression , hunting and gang bangin ‘ genes from dads .

(children also get looks from dads but its only my observation through life)

That is why library girls (and all the girls) want to fuck bad boy but marry providers . And men want to fuck sluts but marry library girls . 😊

74)

Another commenter called Miranda's had this particular line to say, “Women are social creatures and they're designed that way. They talk nonstop and gossip and create social circles

very easily.” They do this because they need to and have always needed to for our entire history as a species. When it comes time to reproduce for a woman, she needs a lot of resources and a stable environment to raise a child. She can't act like a man and wander wherever she wants. This makes women needing to rely on men and their resources to raise the kids and create a stable home for the entire family. And this is why our wallets are so attractive to them. They long to do what we say and desire to have a strong man in their lives. That is why male-only clubs are picketed and have so much hate. Women have an extremely strong programmed need to belong and be wanted. When we say we no longer want to have anything to do with them, or to basically become MGTOWs, it shows them a couple of different things. N°1, it rejects them: due to their higher desire to be social creatures, women can't handle rejection very well. They need to fit in with the people around them. And two, it shows us as flawed males in their eyes. What kind of a man wouldn't want to be with the woman? And according to them, that shows that those men must be betas and should therefore be destroyed. No woman can tolerate a weak man”. ‘ (citing a redditor).

75)

Why does Schopenhauer indicate women reach the maturity of their reasoning and mental faculties at the age of eighteen? Well, what would be the evolutionary advantage to women growing out of this phase of relating to children and beyond it? There is none.

76)

Men's attraction to nice women may be explained by men's desire to ensure their paternity making them prefer well-behaved and controllable women, if they should find them.

77)

One of the common motivators of vegetarian men in adopting such a diet is connected to altruistic concerns (i.e. regarding animal welfare), but in women's average opinion this is sexually unattractive behavior, largely due to perceptions of vegetarian men as being less masculine. Women's preference for omnivorous men might be partly due to the easier digestibility and nutritiousness of meat and the fact that men conducted the vast majority of hunting throughout human evolutionary history, as women are substantially less adapted towards hunting than men.

78)

An alternative or additional explanation may be sexual selection: Geoffrey Miller's mating mind hypothesis of human intelligence suggests that higher cognition and much of human behavior may have evolved as "cognitive ornament" though positive-feedback processes in sexual selection. Such runaway selection predicts not only an exaggerated ornament but also a highly narrowed and exaggerated preference for such ornament.

[…]

The natural aversion to subtle differences in emotional expressiveness may also be a driver of racism as each race may more attuned to particular cognitive ornament specific to their race. For example, Albert Einstein perceived the Northeast Asians as obtuse (ht

tps://www.theguardian.com/books/2018/jun/12/einsteins-travel-diaries-reveal-shocking-xenophobia) in one of his travel diaries.

79)

An awkward woman may also be perceived as neotenous by men as awkwardness is a childish trait. However, neoteny is sexually attractive to men as men like to monopolize women at child age to ensure paternity. Socially awkward women may also seem like "easy prey" for men (Juggernaut law).

80)

Discussion: This result can be regarded as evidence for the sexy son hypothesis, proposed by statistician and geneticist Ronald Fisher (1930). His theory— expanding upon Darwin's much overlooked emphasis on the sexual selection for male traits by females—states that beauty may have evolved by a feedback loop (Fisherian runaway) to become so attractive that women are readily willing to copulate with a beautiful male irrespective of other considerations (e.g. his ability or willingness to provide for and protect the female), because the males' beauty—which is partly heritable—confer on their offspring a potential reproductive advantage. The same does hold true for the opposite case i.e. men more readily copulate with beautiful women, but men can afford to be much more less selective/more promiscuous in any case because they do not need to pay the cost of carrying and giving birth to the child and do not need to consider women's ability to provide (Bateman's Principle of differential parental investment). Hence, women's behavior of disregarding the ability to provide merely at the benefit of better looking offspring has much more drastic implications.

81)

People like us go years and years without sexual release and that signals to our body that they are not evolutionarily successful so we become subordinate in the sexual dominance hierarchy and produce much less testosterone. When the opposite happens and Chads have lots of sex, their testosterone levels rise significantly as their bodies perceive them as evolutionarily successful. This is because every humans evolutionary goal is to create as many children as possible. Therefore, a chad who has sex many times will be signalling his body how successful he is and his body will change because of this. For us it is the opposite. ITS AN UNESCAPABLE LOOP

82)

Presumably, adaptations for intrasexual competition drive much of human conflict. People go great lengths to save face, e.g. declare wars, which is presumably primarily an adaptation to evade intrasexually competitive gossip and maintain status. Such adaptations can explain accusations of gayness and other paraphilias and related forms of reputation denigration, which may even be a contributing factor in e.g. contemporary rape hysteria.

83)

Women's preference for flowers may come from their natural sexual strategy to arouse sexual attention from men by conspicuous advertisement which can be enhanced by flowers which fulfill a similar function in attracting insects by low-entropy signals (sweet scent, bright colors etc.). Dancing also increasingly seems to serve women's sexual advertisement,

displaying thighs, buttock and other aesthetically selected traits. Female adult crying is an aspect of neoteny.”

84)

This impression of plant eating men as less masculine is possibly a carryover of deep social history where the most proficient hunters brought home the game and had higher ranking of choice not only of the meat available to the tribe, but of females. Hunting required strength and prowess. The male gatherers of a tribe in most cultures were seen as low class, often the elderly and infirm.[*citation needed*] Moreover, the most successful hunters could typically best attract women and had more offspring, even in the most egalitarian hunter-gatherers like the Ache and the Sharanahua.

85)

A man’s tendency to bully may also be subject to Fisherian sexual selection. As the tendency to bully is substantially heritable, women may be attracted to this behavior solely for the reproductive benefits it may potentially confer to her male offspring.

86)

Furthermore, I realized women were actually more obsessed with sex than men, in that men do not analyze the sexual implications of every situation, hence they devote more brain power to philosophy, history, art and mathematics, whereas it seems like women constantly think of sex to some degree and interpret every situation in terms of its sexual implications (this may also be because rape was a common feature of human evolution).

87)

What is more, arranged marriage is even more common than free mate choice with 70% of hunter-gatherer societies primarily practicing this kind of marriage (especially in the first marriage). This raises the question whether humans have evolved to desire to be coupled by their parents, which in turn could also explain the rise of modern inceldom in an extremely free mating context. However, such adaptations are difficult to prove scientifically and may fall victim of the naturalistic fallacy.

88)

Indian and Chinese men weren’t natural selected to be beautiful. Arranged marriages mean every bald 5”4 guy with a tiny dick can bass on his genes just as easily as the 6”2 Chad. That’s all well and good but when you remove that system and expect these men to compete with men who were naturally selected to be beautiful (white guys)… Well it’s never be good for the Indians and Chinese.

89)

Interestingly, although the annual incidence of rape for women and being forced to penetrate for men are similar, a much smaller percent of men than women reported either type of victimization over a lifespan. Mathematically this is only possible if a small group of men are being victimized repeatedly, and thus perhaps this provides evidence for hypergamy even in women’s choices of which men they sexually victimize.

90)

Original Just-So Stories Repository

What is presented you is the evidence for women's unsatisfiable appetite for Chad's feces, which promptly called the “poopill”. The poopill virtually overlaps with all known blackpills. For example, the voicepill, a well known fact that women are attracted to men with deep voices. If one has GERD, the constant exposure of stomach acids causes a callus to develop in the throat. This results in many patients with almost unnaturally deep voices. Lack of these protective effects of estrogen in men could possibly explain the higher prevalence of reflux esophagitis in men than in women.

There are gender-related differences in esophageal acid exposure among men and women. Women have significantly fewer reflux events at both esophageal measuring spots, and significantly less total reflux time and percentage of time with pH < 4 in a study for subjects without reflux symptoms or GERD. These results have implications with regard to men’s higher prevalence of BE than women while women have lower esophageal acid exposure in comparison to men.

https://pmc.ncbi.nlm.nih.gov/articles/PMC5056567/

In other words, women may not be necessarily attracted to deep voices in itself, rather, the indication that Chads might have exceedingly high levels stomach acid which will assist with digesting an excessive amount of food. Food that would be processed into ordure.

https://www.sciencedirect.com/science/article/abs/pii/S1090513810000681

When examining masculine features, women selectively find men's mandible particularly attractive:
Follow-up analyses examined the three-way interactions when the individual components of the facial masculinity composite were used instead of the composite. Both effects were in the same predicted direction. The effect involving the three-dimensional, on-site assessment of jaw masculinity was statistically robust,F1,66=4.35,p=.026, partial eta=−0.25.

How does this relate to any of this? A durable jaw is essential to pulverize food into digestible portions. A more pronounced jaw would indicate higher capacity and larger volume for consuming food, which will lead to more fecal quantity.
Two hundred n on-fixed adult cadavers (100men, 100 women) who willingly gave their bodies for scientific purposes were studied. The post mortem average length of the whole intestine was 795.5±129 cm and was significantly longer in men and in young subjects. It was correlated with the subject’s weight but not height.

The average weight was 66±14 kg for the sample with extremes at 40 and 150 kg (74±13 kg for men,58±12 kg for women) (P<10–4). The average height was 164±9 cm for the sample with extremes at 140 and185 cm (170±6 cm for men, 156±5 cm for women)(P<10–4). Click to expand...https://link.springer.com/article/10.1007/s00276-002-0057-y

Men, even when the height are identical cm to cm, are generally larger than their female counterparts, so likewise, their the whole intestine will be larger than their female counterparts. This is another sexual dimorphic trait women have selected for fecal production.

This assumption is reinforced with the fact testosterone assists with gastrointestinal motility.

[testosterone] is essential for maintaining motility in your colon, which helps food move through your digestive tract in a healthy time period.

https://www.atgastro.com/importance-of-testosterone-for-proper-digestive-health-brooklyn-ny/

For example, Fedak et al. ('74) show that human running is 75% less efficient than human walking, so that it is not surprising that comparisons of humans with quadrupedal mammals at walking speed demonstrate greater relative efficiency for the human than comparisons at running speed; and Tucker ('75, Fig. 2) shows that human walking is not energetically expensive relative to true quadrupedalism.

https://onlinelibrary.wiley.com/doi/10.1002/ajpa.1330520113

Why would humanoid females, the gatekeepers of human sex, since their conception would select such males who are so inefficient with basic survival skills such as running? Because the inefficiency would result in the need to consume more substances. The additional consumption will result in more waste, alluring the female's sexual desire. And men ran before the late industrial revolution.

One of the most important biological component for bipedal movement is the gluteus maximus (GM).
The GM as a whole is approximately 1.6 times larger relative to body mass in humans compared to chimpanzees. Dissections indicate that the GM comprises 18.3% of the total mass of the hip musculature in humans, compared to 11.7% and 13.3% for chimpanzees and gorillas, respectively

https://dash.harvard.edu/bitstream/handle/1/3743645/2143.pdf?sequence=1

For the longest time, men assume that women desire massive penises for sexual activity, but if you know anything about scientific research, you'd also know that correlation doesn't mean causation. In this case, women may want men with large phalluses not for themselves rather, because they indicate an above average gluteal mass.

The mean penile length was 13.37 cm with a median of 13 cm and a range of 7.5-19.5 cm. The mean circumference of the body around the buttocks was 96.46 cm (SD = 10.91), median 98 cm, and range 73-122 cm. The body-mass index ranged 17.34-44.44, with the mean at 26.87 (SD = 5.86), and the median 25.53. Linear regression statistics showed no statistically significant correlation between stretched penile length and body-mass index, thus physique. There was a significant direct correlation between penile length and gluteal size.

The supposed relationship between penile length and gluteal size may have a scientific basis, but contrary to belief, large buttocks is more predictive of longer penile length than small buttocks.
Click to expand... https://pubmed.ncbi.nlm.nih.gov/17191423/

Women reported preferring larger obliques, followed by glutes, abdominals..
1629380186600.png

That attractive women generally preferred more developed muscles suggests that women who are higher in mate value are more discriminatory in assessments of bodily attractiveness—a finding that parallels attractive women's higher standards for a variety of potential mate qualities. However, the correlation between SPA and size preferences did not hold for each individual muscle. Women's self-perceived attractiveness was uncorrelated with size preferences for the obliques, abdominals, glutes, and cuadriceps. Click to expand...https://journals.sagepub.com/doi/full/10.1177/1474704919852918

As you can observe women lusts for abs (& muscles surround them) and glutes. In our modern day where physical activates aren't required for obtain food, the only practical value for these myocyte concentrations are for abdominal constriction and sitting. Sit where? You may ask-the toilet. Women wants men who know their shit. Plain and simple.

This is confirmed with the sexual selection that resulted men's muscle concentration in relation to their glucose metabolism.

Genes with men-biased expression also showed enrichment in glucose metabolism path-ways, but the gene sets differed, suggesting alternative pathways in glucose metabolism between men and women (Additional file 14: Table S5). A muscle-contraction pathway was also associated with genes overexpressed in men (Additional file 14: Table S5).This might be related to the physiological differences in muscle tissues and in physical features between men and women.

https://bmcbiol.biomedcentral.com/articles/10.1186/s12915-017-0352-z

Enough of the eyes (and maybe taste), what about the nose? Women's olfactorial sensitivity is heighten during ovulation, thus thus their desire for pheromones is also peaks during ovulation.

Udry delineated the relation between coitus, orgasm and position in the menstrual cycle demonstrating that women engage in sexual intercourse about six times more frequently and are much more likely to have an orgasm at the time of ovulation. During and in the 2–3 days after menses, they were several times less likely to have sexual intercourse or have an orgasm. Coupled with women's odour sensitivity, these results could indicate a possible pheromonal trigger for sexual behaviour. Click to expand...https://sites.oxy.edu/clint/physio/article/PheromonesinsexandreproductionDotheyhavearoleinhumans.pdf

Have you ever wondered where these male pheromones are produced (besides the armpit)? most research on pheromones in humans indicates that the main odor-producing organ is the skin. For the purposes of this paper, the skin is what I will focus on. These odors are largely produced by the skin's apocrine sebaceous glands, which develop during puberty and are usually associated with sweat glands and tufts of hair. These glands are located everywhere on the body surface, but tend to concentrate in six areas: 1) The axillae (underarms) 2) The nipples of both sexes 3) The pubic, genital, and circumanal regions 4) The circumoral region and lips 5) The eyelids 6) The outer ear

Original Just-So Stories Repository

The six areas outlined here are generally found in caucasians, but blacks and Aborigines tend to have more and larger glands, with a higher number on the chest and abdomen than is found in an average caucasian. In addition, Aborigines have a much more powerful scent gland in the circumanal region. Asians, on the other hand, tend to have smaller and far fewer apocrine glands than either Caucasians or blacks, and many have none at all.
Click to expand...http://www.anapsid.org/pheromones.html

The circumanal region is around the anus. Around the same location where one defecates. Also this pill overlaps with the racepill.
Besides pheromones, what also might give circumanal odor their smell?

42 male odour donors were allocated to either a “garlic” or “non-garlic” condition, after which they wore axillary pads for 12 h to collect body odour. One week later, the conditions were reversed. Odour samples were then judged for their pleasantness, attractiveness, masculinity and intensity by 82 women. We found no significant differences in ratings of any characteristics in study 1. However, the odour of donors after an increased garlic dosage was assessed as significantly more pleasant, attractive and less intense (study 2), and more attractive and less intense in study 3. Our results indicate that garlic consumption may have positive effects on perceived body odour hedonicity, perhaps due to its health effects (e.g., antioxidant properties, antimicrobial activity).
Click to expand...https://www.sciencedirect.com/science/article/abs/pii/S0195666315300787

Garlic and onions are top offenders that make things more odorous, as they contain sulfates, which break down into smelly substances.

https://www.livestrong.com/article/13729420-why-does-my-poop-smell-so-bad/

Together, hydrogen sulfide, methanethiol, which smells of rotten cabbage, and garlic-like dimethyl sulfide on average comprised just 50ppm of each fart.

https://www.chemistryworld.com/news/explainer-the-chemistry-of-farts/2500168.article

So it's actually the concentration of methanethiol that causes female specimens to be repealed to the 80% of males, in other words, it's the despite of that particular stench make women attracted to Chads' excrement & flatulence (from the sulfur).
The microbiome plays a vital role with the production of these sulfides. The population density of certain gut bacteria so happens be sexually dimorphic, which means women selected men because of this smell.

Gender-specific differences (P = 0.036) in the Bacteroides-Prevotella group (Bac303) were observed in the total study population with higher levels in males than in females.

https://journals.asm.org/doi/10.1128/aem.72.2.1027-1033.2006

Among these sex-related bacteria, three species were from Clostridia, one from Bacteroidetes, and two from Proteobacteria. All of these species had higher abundance in males than in females.

https://pmc.ncbi.nlm.nih.gov/articles/PMC2538887/

It's not a coincidence that after the sexual revolution started, women would engage in anal foreplay eventually.
Twenty-four percent of guys had performed anilingus on their female partners, while 15 percent had received it. (Another 24 percent had been anally fingered.)

https://www.psychologytoday.com/blo...2/heterosexual-anal-play-increasingly-popular

The Cosmopolitan magazine conducted a poll where the company ask over two thousand women their sentiment on men's decrement. Revealing not only they universally had an opinion on this matter (perhaps all female sapiens did since the dawn of time) also they prefer men who create huge dumps. Their minds must have been set before the survey, otherwise they would've been offended and refused to answer.
teddit.net/r/ShitCosmoSays/comments/7mmycf/reader_poll_about_womens_preferences_in_the_size/

If the poopill model is correct, then we should observe/predict correspondence with it and women's personal activities. Indeed we do see them, even in history.

Why would women desire male brownies? Perhaps even before the hunter-gathering days, females made the connection between crop production and fertilizer. After this discovery, females gave food (w/ seeds) to the males who tend to venture outside the group (usually for game and territory expansion) which they'll defecate wherever these guys journeyed, and the females bred with those males. (Creating the Chad we know today). This scheme resulted in larger territories where both more crops to gather and dung to enjoy. This further produced an evolutionary domination over other sapiens which caused a selection of females who lusts for Chads' fecal matter.

Everything we know about female attraction regarding voice, looks, height (technically weight), etc. traces back to women's sexual compulsion for Chad's manure. Women's main anatomical preference of men deals with digestion. The pheromones and garlic-like sulfides that women find sexually alluring surrounds men's anuses. Women selected men with more gut bacteria, which will assist with guano production. And the cultural and historical evidence only reinforces the model that is the poopill.

91)

Men's fetish for lesbians and cute, playful weak women may also come from this, as wives who get additional sexual pleasure from each other rather than from other men would have been preferred. Of course all of this once again boils down to Bateman's principle and women specializing on child care and extracting resources from men.

92)

Women's emotional instability may also result from their higher reproductive success due to men more readily overlooking flaws in character in order to get laid at all.

93)

In the discussion of a paper revealing that more women were involved in cases of mass hysteria:

« This sex difference may in part result from women's resource dependence. Acting in a dependent, helpless manner allows them to manipulate others into providing resources to them. It may also result from men preferring obedient women as those are less likely to cuckold them, which matters since resource investment is so expensive for men.

94)

The twerk is claimed to have originated from the bounce music scene of New Orleans in the late 1980s. But being such a primitive movement, its origin rather lies in *mammalian lordosis behavior* (Greek lordōsis, from lordos "bent backward"), namely the presentation of the "tail" with upward curved spine to signal receptivity to copulation found in many mammals.[1] Scientists claim human lordosis is only a *vestigial remnant* of proceptivity-/receptivity-communicative signal between male and female,[2] but eye-tracking reveals the arched back does catch both women's and men's attention,[3] also explaining why women wear high heels as it optimizes the "lordotic" posture.[4][5] Mammalian lordosis may have an even more ancient root in reptile behavior in which the female submits itself to the most dominant male.[6][7]

Being a rhythmic movement, the twerk must have evolved in the context of sex orgies as a group of musicians would have been necessary to play the drums which would also arouse the attention of nearby men.

The human preference for shaky buttocks may as well have been sexually selected, causing men to literally have a jiggle physics detector in the hindbrain right next to the penis control area.

95)

The ability to rape may also act as an honest signal (https://en.wikipedia.org/wiki/Signalling_theory) of physical strength and high status. Alternatively (though these two things are of course not mutually exclusive) such tendencies may be reinforced by fisherian runaway sexual selection feedback loops, as the traits that predispose a man to raping are likely substantially heritable. So selecting for a man with 'rapist genes' would ensure that her male offspring inherit these genes, which would thus increase said male offspring's chance of becoming polygynous (in certain opportunistic contexts) which would serve to increase her fitness in an evolutionary sense. Women's general reluctance to have sex and wish to be forced into sex may also test men for their physical strength, as women depend on a physically strong man to be protected, e.g. from other contenders (bodyguard hypothesis). This is related to the male dominance/female surrender pattern that is common in the animal world. The male must present a display of dominance, continue pursuing the female even in the face of rejection, and sometimes even physically subdue the female coerce her into sex (Fisher, 1999). This is possibly a test of his power, fitness, and status. Fisher also suggests that females may have a natural desire to surrender to a pre-selected, dominant male. Eibl-Eibesfeldt (1989) suggests this behavior derives from primitive brain regions that have evolved to insure successful mating in reptiles, birds, and mammals. The fact that many or even most women desire to be dominated reminds one of certain redpill insights as it is actually something men can arguably improve on. However, it remains a blackpill insofar as men are continually heavily shamed by feminists and risk being accused of sexual harassment for their attempts at dominating a female. Due to their evolutionary history, women are also likely very sensitive to false signals of male dominance or status which would make the mimicry of such behavior even riskier. Such a cultural practice is also arguably dysgenic in the sense that it appears to select for psychopathic, impulsive, or just plain unintelligent men who either don't care about such

shaming or lack the knowledge of social norms that would restrain them from behaving in this fashion.

96) (on women committing more Intimate Partner Violence than men)

Another explanation would be that women arrest in their emotional development in their mid-teens, are thus more neotenous, thus more likely may suffer childish anger tantrums. Women also score higher than men in neuroticism (Kajonius, 2018). Women's neotenous neuroticism and anger may be a sneaky adaptation to ensure that they get fed. Women may also test their partner's strength this way.

97)

The higher sexlessness among east Asians compared to other races found by this survey may be related to several factors, cultural and biological. The greater level of physical neoteny found among Asians benefits Asian women, as men have a large preference for neoteny in women. However, it may be a detriment to Asian men's physical attractiveness, resulting in men of other races preferring Asian women. This leads to Asian men losing out as many of their women prefer to date men of other races, especially whites. Asians are also generally higher in the big-five personality traits extraversion and neuroticism than other racial groups, and this may be a detriment especially to Asian men's courtship chances due to women's passive courtship style. There is a common stereotype that Asians are polite, timid and passive, traits that generally seem detrimental to the sexual success of men in particular. It is also possible that a slower overall life history speed among Asians is associated with higher levels of sexlessness in general, with this slower life history speed resulting in later physical maturation and onset of sexual behavior, as well as involving concomitant psychological adaptions that result in deferred reproduction in return for larger investments in somatic effort (biological, phenotypical and in humans, economic investments in and by an organism that result in greater reproductive potential later in life)

98) (about Asian women in the US having the highest rate of exogamous marriage)

This phenomenon may in part be explained by East Asian women's high degree of neoteny acting as a super stimulus to men of other races.

99)

The only reason we aren't non sentient NPC animals is because beta cavemen attacked Chad's authority at one point and fairly distributed the foids under the condition that one will contribute to the tribe/community/society. This was undone by either the lite of the nosetribe or white germanic europeans/descendants of them (five eyes nations). Probably because of monetary gains and more social control over their slaves (basically everyone else).

100)

The authors interpreted their results as evidence that women's choice plays a surprisingly minor role, but another possibility is that women's choice is more based on dominance status than women would like to admit (virtue signaling, social desirability bias).

Overall, this study supports the notion that due to the decline of tradition in Western countries, mating has reverted from more k-strategies that involve sophisticated courtship in terms of impressing not just the female, but gaining competence status in some communal

structures, to mere physical intimidation games, which may in part explain the ever growing interest in body building and anabolic steroids, presumably resembling more the mating practices of our more r-selected ancestors, and also reviving ancient, feral and hypergamous circuitry in women.

101) (About a study which found that women rapidly lose interest in sex once in a stable relationship or living with a man)

A number of potential explanations could be provided for women's faster decline in sexual interest:

- Women's sexuality may be stuck in a more r-selected past because they have been subject to less selective pressure, having had a higher reproductive success throughout human history.
- Different from men, women have a very passive sexuality. Women do not need to compete with costly resource accrual or physical intimidation in order to attract a new mate, but only compete in comparably petty dimensions such as beauty and reputation. As a result, mate switching is associated with considerably lower cost for them compared to men, which might make it worth while to try to get a better man after a while, especially as a good beta provider may even invest in her prior offspring. This kind of sexual conflict is also discussed on the slut article. Corroborating this, women have typically more sexual opportunities than men as men have a more active sexuality, e.g. in online dating they receive around 8 times as many messages, making their cost to switch even lower.
- *Mate switching hypothesis* by Buss (2017), i.e. that switching is facilitated by a number of behavioral adaptations that allow women to leave poor relationships and get access to potentially better partners, implement exit strategies, and manage challenges confronted in the aftermath.

- Women desire testing other men for being potentially more dominant than their current partner so as to be always attached to the most dominant man at all times (bodyguard hypothesis.

- Another explanation may be that the higher choosiness in women simply makes them more aware of flaws such that they simply become dissatisfied sooner, which become especially salient with deep intimacy resulting from cohabitation.

- Women may be somewhat adapted to other harem wives being around, often living in a separate accommodation, so the mere cohabitation with their husband may be an evolutionary mismatch.

- Dissatisfaction, boredom and fickleness are neotenous traits which men select for.

- Men have a greater incentive to keep a sexual monopoly over their female partner for paternity assurance.

- Some argued women's sexual competition primarily lies in competing by arousing men's attention to get access to resources. Once their partner of interest is on the verge of

showing disinterest, women become anxious and will readily offer sex to secure his resource investment and protection. Some argued, women engage in this behavior as disinterest on part of the male is a costly signal of having alternative options and this observation lead to the invention of the "dread game" in redpill circles, meaning the deliberate display of disinterest to provoke women's voluntary offer of sex. In a long-term bond, womens gain certainty to have secured her husband's resources, so they see less need for rewarding her partner with sex. If the man is high, status, however, he has a multitude of alternative options, so she continuously needs to secure his resources with sex. This is also corroborated by Klusmann's study as it found that when women's status (in terms of educational status) is lower than the man's then she does not lose her sex drive.

102)

Discussion: This study provides some support for the 'exploitation hypothesis' of women's attraction to Dark Triad traits in men, as the women in the study were generally averse to men displaying overtly antisocial traits, evaluating these men more unfavorably in a romantic context. This suggests that the fake pro-social, glib, superficially charming aspect of psychopathy is what the women found most attractive. However, the women in the study also responded more favorably to the men with higher levels of affective psychopathy (i.e., those who demonstrated lower levels of empathy and callous behavior) on an innate, subconscious level (higher vocal pitch when leaving a voice message for them). This suggests that women may also strongly favor some of the more overtly socially undesirable psychopathy aspects in a romantic context. The 'lifestyle' aspects of psychopathy were also evaluated favorably by the women in the study, especially when this evaluation depended on the women's conscious, subjective rating of the men. These 'psychopathic lifestyle' traits include lack of clear life goals, socially parasitic behavior, and irresponsibility, not characteristics that would make these men good providers or prone to commit to long term relationships. The women's preference for psychopathy's lifestyle aspects may stem from these traits being associated with (at least on the surface) a fun-loving, laid-back or adventurous nature, and a general lack of social inhibition. Effectively personality traits that would keep the women constantly emotionally More psychopathic men tend to receive higher attractiveness ratings from women stimulated and prevent her from being bored in the relationship. As women were generally dependent on men for provision throughout their evolutionary history. It could be that women only care about traits that would make men good providers for long-term relationships, perhaps even evaluating them negatively in shorter-term relationships. It could also be that these provider traits were not directly sexually selected at all, and women themselves did not choose these traits throughout history. However, their parents likely selected these traits in men (with these traits being associated with socio-economic success and reliability). Historically, a substantial portion of marriages were arranged by women's parents. The lack of a strong female preference for the overtly antisocial aspects of psychopathy, such as aggressive behavior, indicates that these traits may have been evolutionarily selected by allowing ancestral men with these traits to prevail in male-male contests, rather than through a direct female preference for such characteristics. One would suspect men prone to using violence or the threat of it to be more successful in deterring potential male rivals (such as mate-poachers). These violent tendencies would also be expected to aid men in ascending social hierarchies based primarily on dominance rather than prestige, by allowing them to survive and acquire resources and higher social status that would have

assisted them in attracting women (directly or indirectly) and being able to pass on their genes. This study seemingly indicates a female preference for men that are unsuited towards longer-term relationships. This preference, no matter how slight it may be, seems to provide some support to arguments that many modern women are making maladaptive mate choices due to an evolutionary mismatch between historical and contemporary mating contexts. Of course, one could argue this also applies to men as their mate choices were also often constrained in the past, though the consequences of possible spousal abandonment would be far less harsh for them.
