# Supplementary material for: A hundred and two just-so stories: exploring the lay evolutionary hypotheses of the manosphere
Source: Evol Hum Sci. 2025 Oct 9;7:e41. doi: 10.1017/ehs.2025.10020 (PMC12645320; doi:10.1017/ehs.2025.10020)
Supplement: Bachaud et al. supplementary material [file S2513843X25100200sup001.zip › S2513843X25100200sup001/Supplementary Material S9.pdf]

Coding Instructions

Sexual Selection: If a trait evolved because it made individuals (or their offspring) more reproductively successful by outcompeting rivals/favoring them in mate choice, (this includes sperm competition/selection after copulation).

Careful: this is just a subset of natural selection, so one should be conservative in coding something as "sexual selection." Anything that increases health, prestige, dominance, etc. presumably ends up having downstream reproductive advantages (this is after all the engine of natural selection), but only straightforward "direct" cases of sexual selection (where mate competition/mate choice is explicitly designated as the main evolutionary driver) should be selected here.

Code: Yes = 1; No = 0.

Evolved Sex Difference: Does the adaptationist hypothesis include (a) different selective pressures weighing on males and females, and/or (b) a resulting sex-differentiated adaptation (usually both go together). In other words, did the evolutionary process hypothesized involve differential selection of genes between males and females, resulting in different genetic underpinning of behavior?

(note: could of course be different gene expression rather than different genes between the sexes, the mechanisms could be varied, hormonal, cognitive, often unspecified).

Code: Yes = 1; No = 0.

Marks of hypothesis: Did the manospherian formulating the Just-so story acknowledge the speculative nature of the JSS? Could come in several forms:

- Adverbs like "maybe" or "potentially"
- Modals like "may" or "might"
- Calling his JSS a hypothesis
- Using a sentence to express uncertainty "I guess"

Careful: the presence of just one of these words/phrases in the JSS should not automatically imply that there is a mark of hypothesis about the JSS itself. The mark of hypothesis should concern the core adaptationist explanation for the trait (i.e., A evolved because of B). So, you should consider things which fundamentally mean: "A evolved because maybe B"; or "I guess A evolved because B"; or "A may have evolved because B".

Code: Yes = 1; No = 0.
