# Supplementary figures and images for: A hundred and two just-so stories: exploring the lay evolutionary hypotheses of the manosphere
Source: Evol Hum Sci. 2025 Oct 9;7:e41. doi: 10.1017/ehs.2025.10020 (PMC12645320; doi:10.1017/ehs.2025.10020)

Figure S4: Manosphere Corpus Material Selection Process

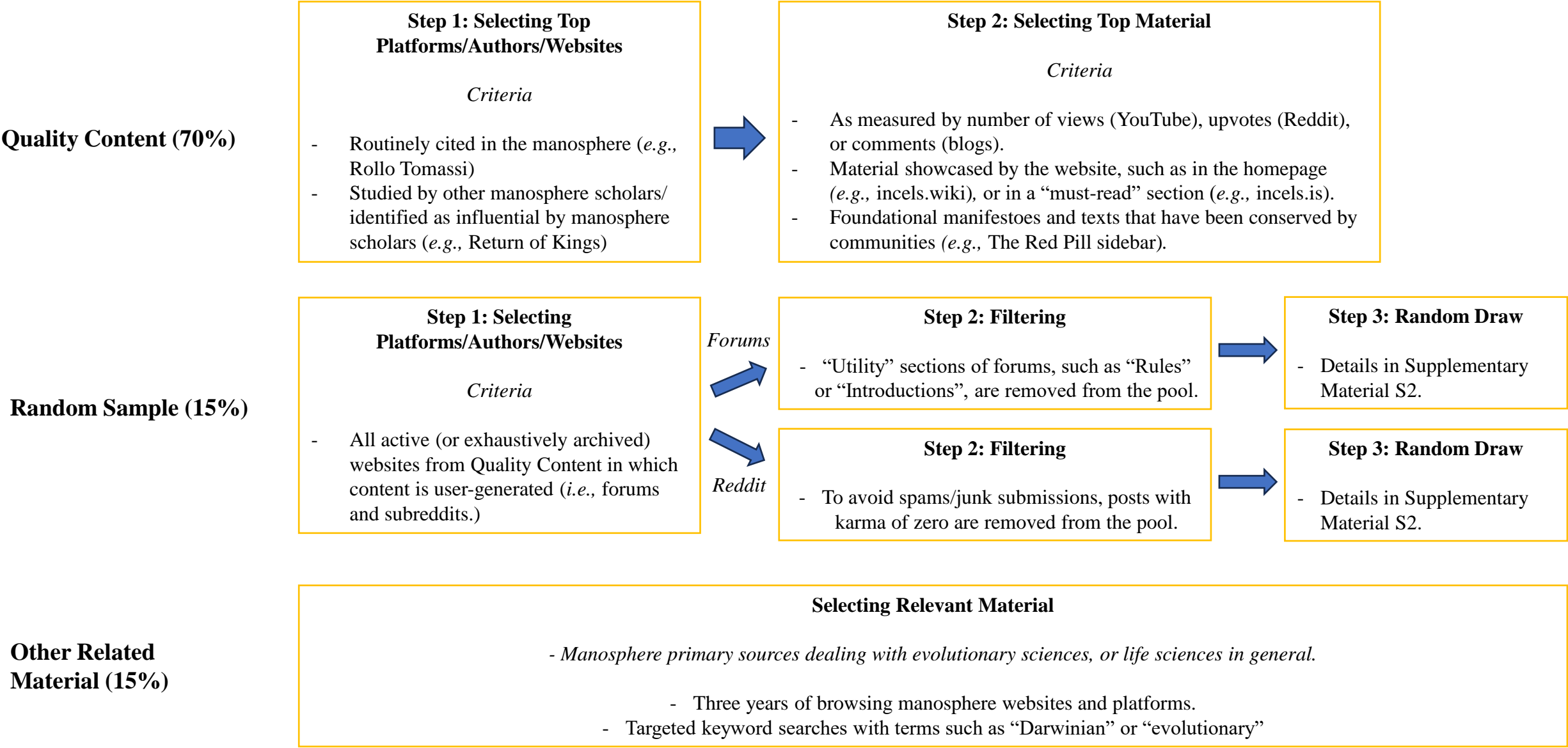

Supplement: Bachaud et al. supplementary material [file S2513843X25100200sup001.zip › S2513843X25100200sup001/Supplementary Material S4.pdf]

## Supplementary Material S8: Just-So Stories Identification Process

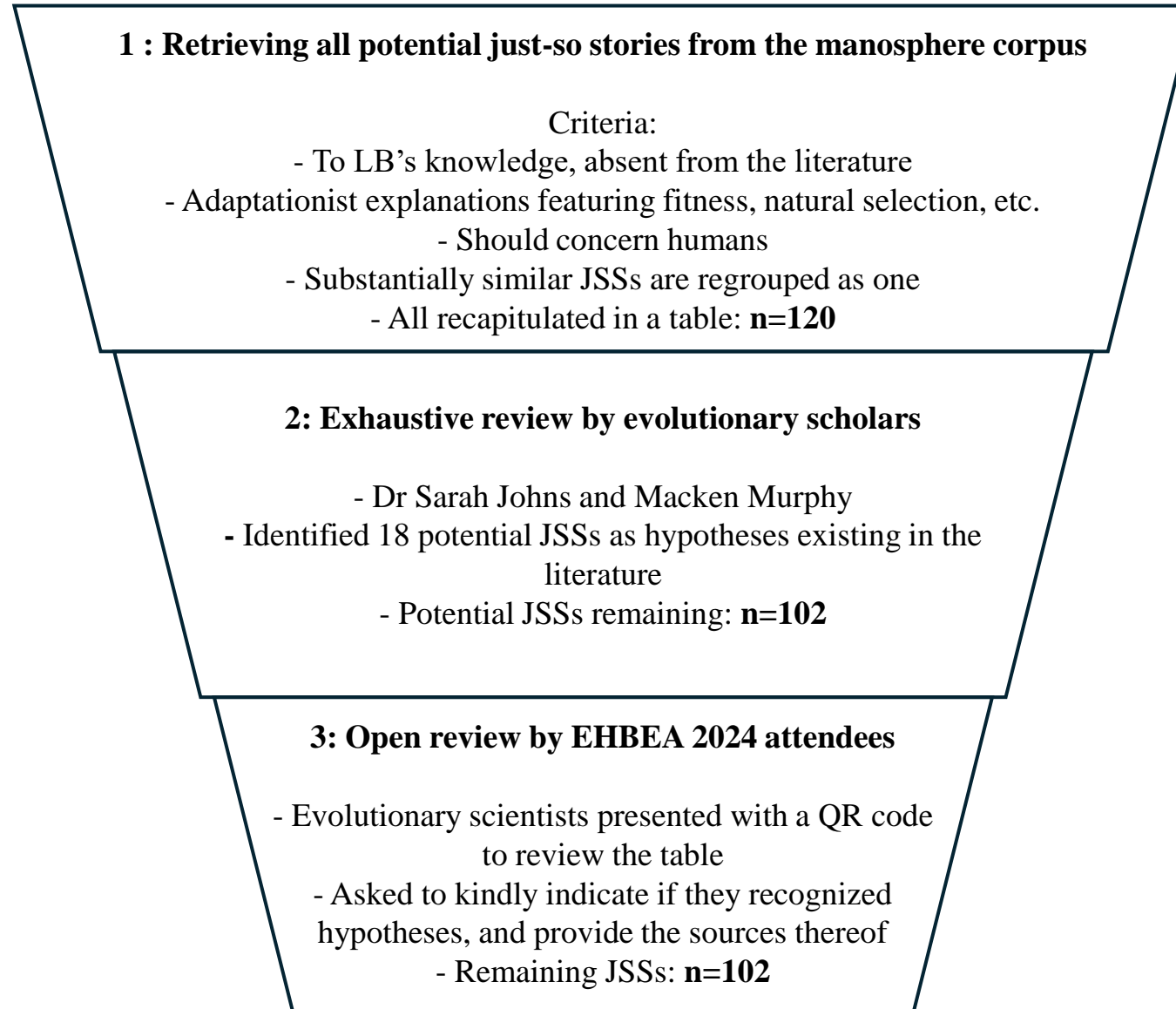

Supplement: Bachaud et al. supplementary material [file S2513843X25100200sup001.zip › S2513843X25100200sup001/Supplementary Material S8.pdf]
